# Comparative Long-Term Clinical Performance of Mechanical Aortic Valve Prostheses

**DOI:** 10.1001/jamanetworkopen.2024.7525

**Published:** 2024-04-19

**Authors:** Malin Granbom Koski, Natalie Glaser, Anders Franco-Cereceda, Ulrik Sartipy, Michael Dismorr

**Affiliations:** 1 Department of Molecular Medicine and Surgery, Karolinska Institutet, Stockholm, Sweden; 2 Department of Cardiothoracic Surgery, Karolinska University Hospital, Stockholm, Sweden; 3 Department of Cardiology, Stockholm South General Hospital, Stockholm, Sweden

## Abstract

**Importance:**

Aggregated data and long-term follow-up in national health data registers offer the opportunity to compare the performance of mechanical aortic prostheses within the same population.

**Objective:**

To investigate the clinical performance of mechanical aortic valve prostheses.

**Design, Setting, and Participants:**

This nationwide cohort study included all 5224 patients who underwent primary mechanical aortic valve replacement in Sweden between January 1, 2003, and December 31, 2018. Statistical analysis was performed between May and September 2023.

**Exposures:**

Surgical aortic valve replacement with the On-X, Carbomedics, Bicarbon, Standard, Regent, Open Pivot, Masters, or Advantage valve models.

**Main Outcomes and Measures:**

The primary outcome was all-cause mortality, and secondary outcomes were reintervention, heart failure, major bleeding, stroke, and embolic events. Regression standardization was used to account for baseline differences.

**Results:**

Overall, 5224 patients (mean [SD] age, 56.8 [11.7] years; 3908 men [74.8%]) were included. Total follow-up time was 43 982 person-years (mean [SD], 8.4 [4.6] years; maximum, 17.2 years). After regression standardization, there was a significant difference in 10-year mortality between the Carbomedics model group (17%; 95% CI, 15%-18%), Regent model group (17%; 95% CI, 13%-20%), and Standard model group (17%; 95% CI, 14%-19%) compared with the Bicarbon model group (27%; 95% CI, 21%-34%).

**Conclusions and Relevance:**

In this cohort study of mechanical valve surgical aortic replacement outcomes in Sweden, the rate of all-cause mortality was higher in the Bicarbon group than in the Carbomedics, Regent, and Standard model groups. These findings warrant further research on the long-term clinical performance of the Bicarbon valve.

## Key Points

**Question:**

Is there a difference among bileaflet mechanical aortic valve prostheses in the long-term incidence of all-cause mortality, aortic valve reintervention, heart failure, major bleeding, stroke, and embolic events?

**Findings:**

In this cohort study of 5224 patients who underwent surgical aortic valve replacement in Sweden between 2003 and 2018, the Bicarbon valve had a significantly higher incidence of 10-year all-cause mortality (27%) than the Carbomedics (17%), Regent (17%), and Standard (17%) valves. Otherwise, the performance was generally comparable among the different valve groups.

**Meaning:**

This study suggests that further studies on the long-term performance of the Bicarbon valve are warranted.

## Teaser

This cohort study compares the clinical performance of mechanical aortic valve prostheses in Sweden.

## Introduction

Aortic valve replacement with mechanical valve prostheses has been performed since the 1950s.^1^ However, in the past 2 decades, the use of mechanical valves has decreased in favor of bioprosthetic valves across all age groups.^2,3^ This shift can most likely be attributed to the fact that patients who undergo a mechanical valve implant require lifelong warfarin treatment, which subsequently increases the risk of adverse anticoagulation-related events.^4,5^ In addition, improvements in the durability of biological valves have contributed to their increased use. According to European guidelines, mechanical valves are the recommended choice for patients younger than 60 years,^6^ whereas US guidelines recommend mechanical valves for patients younger than 50 years.^7^

Research on the performance of mechanical valves is often limited to studies examining a single valve series or comparing 2 valve models. However, analyzing a larger array of valve models within the same study population provides an opportunity to identify differences in long-term performance among the different valves. Hickey et al^8^ compared the clinical outcomes of aortic valve prostheses using national registry data from the UK, identifying important differences in performance between valve models. Similarly, Persson et al^9^ compared bioprosthetic aortic valve models and identified clinically relevant differences in long-term performance among the different valve model groups. However, to our knowledge, no comparative analyses have been conducted to date in Sweden to examine the long-term performance of different mechanical valve models. Swedish national health data registries, known for their high reliability and excellent coverage, enable meaningful comparisons between different valve models in the same population over a long follow-up period. Accordingly, the aim of this study was to compare the long-term clinical performance of mechanical aortic valve models and to identify which models, if any, demonstrate deviations in performance, warranting further research or clinical vigilance.

## Methods

### Study Design

This was an observational, population-based, nationwide cohort study. It was performed according to the Strengthening the Reporting of Observational Studies in Epidemiology (STROBE) reporting guideline^10^ and the Reporting of Studies Conducted Using Observational Routinely Collected Health Data (RECORD) statement.^11^ This study was approved by the regional Human Research Ethics Committee, Stockholm, Sweden. The requirement for informed consent was waived because the data were deidentified.

### Study Population

Patients who underwent surgical aortic valve replacement (SAVR) with mechanical aortic valves between January 1, 2003, and December 31, 2018, in Sweden, with or without concurrent coronary artery bypass grafting surgery or ascending aortic surgery, were included. The exclusion criteria were being younger than 18 years of age, undergoing concurrent surgery on another valve, undergoing surgery with the use of deep hypothermia or circulatory arrest, having undergone cardiac surgery or transcatheter aortic valve replacement, use of a tilting-disk or ball-in-cage prothesis, or undetermined prosthesis type.

### Exposure and Outcomes

The exposure was defined as having received a prosthetic mechanical aortic valve. The following valve model groups were analyzed in the study: On-X, Carbomedics, Bicarbon, Standard, Regent, Open Pivot, Masters, and Advantage. A few valve models were excluded due to low rates of use. The frequencies and types of valves within each valve model group are presented in eTable 1 in Supplement 1. The primary outcome was all-cause mortality, identified from the Total Population Register.^12^ The secondary outcomes were aortic valve reintervention (identified from the Swedish Cardiac Surgery Registry^13^); heart failure hospitalization; bleeding; and stroke, transient ischemic attack (TIA), or embolic event, all identified from the national patient register through *International Statistical Classification of Diseases and Related Health Problems, Tenth Revision* codes^14^ (codes used to identify the secondary outcomes in the National Patient Register are presented in eTable 2 in Supplement 1).

### Data Sources

The Swedish Cardiac Surgery Registry, part of the Swedish Web System for Enhancement and Development of Evidence-Based Care in Heart Disease Evaluated According to Recommended Therapies (SWEDEHEART) Register, was used to identify the study population.^15^ The Swedish Cardiac Surgery Registry collects preoperative, perioperative, and postoperative data on all cardiac surgery patients nationwide with high reliability.^13^ The National Patient Register was used to collect baseline characteristics of preexisting morbidities, as well as outcome data for the heart failure; transcatheter aortic valve implant reintervention; bleeding events; and stroke, TIA, and embolic events. The National Patient Register has full coverage of all inpatient diagnoses in Sweden and has high validity.^14^ The longitudinal integrated database for health insurance and labour market studies register^16^ was used to obtain the socioeconomic baseline characteristics of the study population. The Swedish personal identity number enabled cross-linking between the registers.^17^

### Statistical Analysis

Statistical analysis was performed between May and September 2023. Baseline characteristics are described as mean (SD) values for continuous variables. Categorical variables are described as frequencies and percentages. Baseline differences between the groups were described as standardized mean differences as estimated by the R package tableone (R, version 4.1.0; R Project for Statistical Computing). Time to event was determined as the time in days from the date of surgery to the date of event or the end of follow-up on December 31, 2018. A Poisson model was used to estimate age- and sex-adjusted incidence rates. The Aalen-Johansen estimator was used to estimate the crude cumulative incidence while accounting for the competing risk of death. Flexible parametric regression standardization was used to estimate cumulative survival while adjusting for the distribution of covariates in the population.^18^ The resulting survival curve is an estimate of population survival if the entire population had received the same of each respective valve. Flexible hazard-based regression standardization as described by Kipouro et al^19^ was used to estimate cumulative incidence of reintervention; heart failure hospitalization; bleeding events; and stroke, TIA, or embolic events. This method adjusts for the population distribution of covariates while accounting for the competing risk of death. The resulting cumulative incidence curve can be interpreted as the estimated cumulative incidence if the whole study population received the same of each valve model. Model selection was performed using clinical subject matter knowledge and was guided by the Akaike information criterion. Covariates included in the different models are presented in the eMethods in Supplement 1. Missing data were handled with the classification and regression tree estimation and imputation approach (eMethods in Supplement 1).^20^ Data management and statistical analyses were performed using R, version 4.1.0 (R Project for Statistical Computing), including the survival, ggplot, mexhaz, stdReg, and rstpm2 packages.^21,22,23,24,25^ All *P* values were from 2-sided tests and results were deemed statistically significant at *P* < .05.

## Results

We identified 5224 patients (mean [SD] age, 56.8 [11.7] years; 3908 men [74.8%]) who underwent SAVR with a mechanical aortic valve prosthesis in Sweden between 2003 and 2018. There were differences in the baseline characteristics of the patients, including in age, sex, left ventricular ejection fraction, and valve prosthesis size (Table 1). The number of valves implanted per model group and year is shown in eFigure 1 in Supplement 1. The distribution of age, valve size, left ventricular ejection fraction, and sex per model group is shown in eFigures 2, 3, 4, and 5 in Supplement 1.

**Table 1.** Baseline Characteristics of Patients Who Underwent Aortic Valve Replacement With Mechanical Prostheses in Sweden Between 2003 and 2018

Variable	Patients, No. (%)									SMD
	Overall	On-X	Carbomedics	Bicarbon	Standard	Regent	Open Pivot	Masters	Advantage	
Total	5224 (100)	679 (13.0)	2171 (41.6)	164 (3.1)	418 (8.0)	567 (10.9)	231 (4.4)	827 (15.8)	167 (3.2)	NA
Age, mean (SD), y	56.8 (11.7)	52.3 (11.4)	56.1 (10.5)	56.6 (12.5)	69.5 (12.2)	56.6 (12.0)	57.2 (8.4)	55.6 (11.0)	59.0 (9.0)	0.451
Female	1316 (25.2)	122 (18.0)	494 (22.8)	57 (34.8)	145 (34.7)	155 (27.3)	76 (32.9)	226 (27.3)	41 (24.6)	0.164
Married	2791 (53.4)	313 (46.1)	1184 (54.5)	86 (52.4)	253 (60.5)	280 (49.4)	117 (50.6)	458 (55.4)	100 (59.9)	0.122
BMI										
<18.5	36 (0.7)	5 (0.8)	18 (0.9)	3 (1.9)	2 (0.5)	3 (0.6)	1 (0.4)	3 (0.4)	1 (0.7)	0.231
18.5-24.9	1466 (30.4)	192 (29.4)	594 (30.5)	53 (33.5)	153 (39.2)	158 (29.4)	45 (19.6)	237 (31.1)	34 (24.8)	
25-29.9	2029 (42.1)	274 (42.0)	825 (42.4)	54 (34.2)	171 (43.8)	206 (38.3)	107 (46.5)	322 (42.2)	70 (51.1)	
>30	1286 (26.7)	182 (27.9)	511 (26.2)	48 (30.4)	64 (16.4)	171 (31.8)	77 (33.5)	201 (26.3)	32 (23.4)	
Educational level, y										
<10	1605 (30.9)	168 (24.8)	668 (30.9)	42 (25.6)	213 (51.2)	152 (27.1)	57 (24.8)	259 (31.5)	46 (28.0)	0.220
10-12	2429 (46.8)	344 (50.7)	1036 (48.0)	78 (47.6)	141 (33.9)	249 (44.5)	126 (54.8)	374 (45.4)	81 (49.4)	
>12	1161 (22.3)	166 (24.5)	456 (21.1)	44 (26.8)	62 (14.9)	159 (28.4)	47 (20.4)	190 (23.1)	37 (22.6)	
Household income										
Quartile 1 (lowest)	1306 (25.0)	126 (18.6)	534 (24.6)	39 (23.8)	217 (51.9)	107 (18.9)	51 (22.1)	190 (23.0)	42 (25.1)	0.319
Quartile 2	1306 (25.0)	164 (24.2)	553 (25.5)	42 (25.6)	112 (26.8)	130 (22.9)	54 (23.4)	208 (25.2)	43 (25.7)	
Quartile 3	1306 (25.0)	174 (25.6)	575 (26.5)	31 (18.9)	50 (12.0)	137 (24.2)	68 (29.4)	226 (27.3)	45 (26.9)	
Quartile 4 (highest)	1306 (25.0)	215 (31.7)	509 (23.4)	52 (31.7)	39 (9.3)	193 (34.0)	58 (25.1)	203 (24.5)	37 (22.2)	
Non-Nordic birth region	447 (8.6)	35 (5.2)	169 (7.8)	29 (17.7)	19 (4.5)	85 (15.0)	22 (9.5)	78 (9.4)	10 (6.0)	0.184
LVEF, %										
>50	3859 (74.4)	508 (75.0)	1602 (74.2)	123 (75.5)	311 (74.6)	396 (69.8)	184 (79.7)	604 (75.2)	131 (78.4)	0.157
30-50	1059 (20.4)	139 (20.5)	433 (20.1)	25 (15.3)	88 (21.1)	138 (24.3)	39 (16.9)	163 (20.3)	34 (20.4)	
<30	266 (5.1)	30 (4.4)	124 (5.7)	15 (9.2)	18 (4.3)	33 (5.8)	8 (3.5)	36 (4.5)	2 (1.2)	
Prior myocardial infarction	520 (10.0)	49 (7.2)	224 (10.3)	9 (5.5)	80 (19.1)	52 (9.2)	26 (11.3)	66 (8.0)	14 (8.4)	0.142
Prior heart failure	881 (16.9)	101 (14.9)	365 (16.8)	35 (21.3)	87 (20.8)	110 (19.4)	30 (13.0)	128 (15.5)	25 (15.0)	0.097
Prior atrial fibrillation	705 (13.5)	75 (11.0)	279 (12.9)	25 (15.2)	58 (13.9)	106 (18.7)	33 (14.3)	106 (12.8)	23 (13.8)	0.071
Pacemaker or ICD	93 (1.8)	14 (2.1)	31 (1.4)	4 (2.4)	9 (2.2)	13 (2.3)	4 (1.7)	12 (1.5)	6 (3.6)	0.052
Prior PCI	222 (4.2)	26 (3.8)	91 (4.2)	6 (3.7)	15 (3.6)	28 (4.9)	11 (4.8)	35 (4.2)	10 (6.0)	0.045
Hyperlipidemia	853 (16.3)	110 (16.2)	324 (14.9)	40 (24.4)	50 (12.0)	112 (19.8)	52 (22.5)	125 (15.1)	40 (24.0)	0.146
Hypertension	1868 (35.8)	262 (38.6)	688 (31.7)	76 (46.3)	125 (29.9)	250 (44.1)	113 (48.9)	290 (35.1)	64 (38.3)	0.173
Endocarditis	134 (2.6)	21 (3.1)	55 (2.5)	3 (1.8)	10 (2.4)	17 (3.0)	7 (3.0)	17 (2.1)	4 (2.4)	0.036
Peripheral vascular disease	887 (17.0)	100 (14.7)	414 (19.1)	14 (8.5)	66 (15.8)	51 (9.0)	39 (16.9)	185 (22.4)	18 (10.8)	0.170
eGFR, mL/min/1.73 m^2^										
>60	4474 (87.7)	630 (94.6)	1891 (90.2)	145 (89.0)	215 (54.8)	496 (87.8)	218 (94.8)	729 (88.8)	150 (89.8)	0.365
45-59	417 (8.2)	26 (3.9)	122 (5.8)	9 (5.5)	130 (33.2)	44 (7.8)	7 (3.0)	71 (8.6)	8 (4.8)	
30-44	120 (2.4)	4 (0.6)	36 (1.7)	4 (2.5)	38 (9.7)	19 (3.4)	1 (0.4)	13 (1.6)	5 (3.0)	
<30	89 (1.7)	6 (0.9)	47 (2.2)	5 (3.1)	9 (2.3)	6 (1.1)	4 (1.7)	8 (1.0)	4 (2.4)	
COPD	371 (7.1)	26 (3.8)	157 (7.2)	11 (6.7)	38 (9.1)	41 (7.2)	17 (7.4)	64 (7.7)	17 (10.2)	0.081
Diabetes	706 (13.5)	84 (12.4)	272 (12.5)	27 (16.5)	55 (13.2)	102 (18.0)	43 (18.6)	97 (11.7)	26 (15.6)	0.091
Prior stroke	348 (6.7)	40 (5.9)	135 (6.2)	15 (9.1)	34 (8.1)	46 (8.1)	20 (8.7)	43 (5.2)	15 (9.0)	0.070
History of cancer	355 (6.8)	39 (5.7)	125 (5.8)	15 (9.1)	54 (12.9)	43 (7.6)	14 (6.1)	50 (6.0)	15 (9.0)	0.100
Hepatic disease	55 (1.1)	5 (0.7)	23 (1.1)	3 (1.8)	8 (1.9)	7 (1.2)	5 (2.2)	4 (0.5)	0	0.092
Alcohol dependence	147 (2.8)	16 (2.4)	70 (3.2)	3 (1.8)	6 (1.4)	22 (3.9)	12 (5.2)	16 (1.9)	2 (1.2)	0.097
Valve size, mm										
19-21	1157 (22.5)	177 (26.3)	325 (15.4)	69 (42.1)	101 (24.4)	179 (31.6)	57 (24.8)	207 (25.4)	42 (25.1)	0.279
23	1832 (35.6)	300 (44.6)	737 (34.8)	57 (34.8)	129 (31.2)	200 (35.3)	94 (40.9)	255 (31.2)	60 (35.9)	
≥25	2158 (41.9)	195 (29.0)	1055 (49.8)	38 (23.2)	184 (44.4)	188 (33.2)	79 (34.3)	354 (43.4)	65 (38.9)	
Concomitant CABG	1087 (20.8)	101 (14.9)	442 (20.4)	22 (13.4)	207 (49.5)	95 (16.8)	40 (17.3)	137 (16.6)	43 (25.7)	0.268
Ascending aortic surgery	1310 (25.1)	167 (24.6)	677 (31.2)	19 (11.6)	62 (14.8)	67 (11.8)	52 (22.5)	248 (30.0)	18 (10.8)	0.255
Emergency operation	125 (2.4)	17 (2.5)	61 (2.8)	2 (1.2)	10 (2.4)	11 (1.9)	4 (1.7)	17 (2.1)	3 (1.8)	0.043
Period of surgery										
2003-2008	2368 (45.3)	103 (15.2)	1199 (55.2)	19 (11.6)	408 (97.6)	135 (23.8)	48 (20.8)	364 (44.0)	92 (55.1)	1.100
2009-2013	1423 (27.2)	224 (33.0)	470 (21.6)	102 (62.2)	10 (2.4)	189 (33.3)	82 (35.5)	271 (32.8)	75 (44.9)	
2014-2018	1433 (27.4)	352 (51.8)	502 (23.1)	43 (26.2)	0	243 (42.9)	101 (43.7)	192 (23.2)	0	

Abbreviations: BMI, body mass index (calculated as weight in kilograms divided by height in meters squared); CABG, coronary artery bypass grafting; COPD, chronic obstructive pulmonary disease; eGFR, estimated glomerular filtration rate; ICD, implantable cardioverter defibrillator; LVEF, left ventricular ejection fraction; NA, not applicable; PCI, percutaneous coronary intervention; SMD, standardized mean difference.

### All-Cause Mortality

The total follow-up time for all-cause mortality was 43 982 person-years (mean [SD], 8.4 [4.6] years; maximum, 17.2 years), during which 1166 patients (22.3%) died. The crude incidence rate for all-cause mortality per 100 person-years was lowest in the On-X model group (1.6; 95% CI, 1.2-2.0) and highest in the Standard model group (6.7; 95% CI, 6.0-7.5) (eTable 3 in Supplement 1). Age- and sex-adjusted incidence rates per 100 person-years were lowest in the Regent model group (2.1; 95% CI, 1.8-2.5) and highest in the Bicarbon (3.5; 95% CI, 3.2-3.9) and Standard (3.5; 95% CI, 2.7-4.7) model groups.

The crude cumulative incidence of 10-year all-cause mortality was the lowest in the On-X model group (14%; 95% CI, 10%-17%) and the highest in the Standard model group (40%; 95% CI, 36%-45%) (Table 2). The crude cumulative incidence and number of all-cause mortality events at 5, 10, and 15 years are presented in Table 2.

**Table 2.** Crude Cumulative Incidence and Events per PY for All-Cause Mortality, Reintervention, Heart Failure Hospitalization, Bleeding Event, and Stroke, TIA, or Embolic Event After Surgical Aortic Valve Replacement in Sweden Between 2003 and 2018

Characteristic	Events/PY	% (95% CI)		
		At 5 y	At 10 y	At 15 y
**All-cause mortality**				
On-X	65/4184	6 (4-8)	14 (10-17)	23 (16-30)
Carbomedics	470/20 191	8 (6-9)	18 (17-20)	33 (30-36)
Bicarbon	38/1190	14 (8-19)	27 (18-35)	NA
Standard	282/4198	20 (16-24)	40 (36-45)	69 (64-74)
Regent	79/3971	8 (5-10)	17 (13-21)	33 (24-43)
Open Pivot	31/1505	9 (5-13)	19 (11-26)	NA
Masters	155/7106	9 (7-11)	17 (14-20)	35 (28-42)
Advantage	46/1636	13 (8-18)	24 (17-30)	45 (20-70)
**Reintervention** ^a^				
On-X	17/3388	2.3 (1.1-3.5)	3.2 (1.5-4.8)	36.2 (0.0-82.4)
Carbomedics	69/17 712	2.2 (1.6-2.8)	3.0 (2.2-3.8)	4.5 (3.3-5.8)
Bicarbon	1/1029	0.6 (0.0-1.8)	0.6 (0.0-1.8)	NA
Standard	14/3938	1.7 (0.4-2.9)	2.4 (0.9-3.9)	3.9 (1.8-5.9)
Regent	22/3306	2.8 (1.3-4.3)	5.5 (3.0-8.0)	8.6 (3.7-13.4)
Open Pivot	2/1250	0.9 (0.0-2.1)	0.9 (0.0-2.1)	NA
Masters	27/6159	2.3 (1.2-3.3)	3.6 (2.2-5.1)	NA
Advantage	6/1463	1.8 (0.0-3.8)	3.5 (0.4-6.7)	NA
**Heart failure hospitalization** ^a^				
On-X	24/3355	2.8 (1.4-4.1)	6.4 (3.5-9.4)	6.4 (3.5-9.4)
Carbomedics	140/17 619	3.5 (2.7-4.3)	6.5 (5.3-7.7)	10.4 (8.5-12.2)
Bicarbon	12/999	4.3 (1.2-7.4)	9.9 (4.2-15.5)	NA
Standard	82/3746	9.1 (6.3-11.8)	15.3 (11.9-18.8)	21.4 (17.1-25.6)
Regent	34/3253	5.0 (3.0-7.1)	8.6 (5.6-11.6)	10.8 (6.5-15.1)
Open Pivot	10/1224	5.1 (1.8-8.4)	6.9 (2.2-11.6)	NA
Masters	62/6041	4.9 (3.3-6.5)	8.5 (6.2-10.7)	NA
Advantage	13/1443	3.0 (0.4-5.6)	8.6 (4.1-13.2)	NA
**Bleeding event** ^a^				
On-X	36/3309	5.8 (3.7-7.8)	7.6 (5.0-10.2)	12.6 (2.9-22.2)
Carbomedics	220/17 054	6.1 (5.1-7.2)	10.7 (9.2-12.2)	15.3 (13.2-17.4)
Bicarbon	10/1002	5.7 (2.1-9.4)	7.5 (2.5-12.5)	NA
Standard	59/3691	8.4 (5.7-11.0)	11.7 (8.6-14.8)	14.7 (11.1-18.2)
Regent	38/3245	5.9 (3.7-8.1)	9.4 (6.2-12.6)	14.0 (7.8-20.2)
Open Pivot	15/1200	6.0 (2.6-9.3)	9.6 (4.1-15.2)	NA
Masters	63/5984	5.0 (3.4-6.5)	8.8 (6.6-11.0)	NA
Advantage	17/1407	6.0 (2.4-9.6)	9.3 (4.8-13.8)	NA
**Stroke, TIA, or embolic event** ^a^				
On-X	43/3270	7.7 (5.3-10.1)	9.6 (6.7-12.5)	9.6 (6.7-12.5)
Carbomedics	202/17 128	5.6 (4.5-6.6)	9.7 (8.3-11.1)	13.6 (11.7-15.5)
Bicarbon	13/986	6.3 (2.5-10.0)	9.2 (4.3-14.1)	NA
Standard	61/3719	7.4 (4.9-9.9)	11.5 (8.4-14.5)	15.9 (12.2-19.7)
Regent	30/3247	5.5 (3.4-7.6)	7.7 (4.8-10.5)	7.7 (4.8-10.5)
Open Pivot	12/1225	5.6 (2.3-8.8)	6.4 (2.8-9.9)	NA
Masters	57/6014	4.1 (2.7-5.5)	8.1 (5.9-10.3)	NA
Advantage	15/1420	3.0 (0.4-5.6)	8.2 (3.9-12.6)	NA

Abbreviations: NA, not available; PY, person-years; TIA, transient ischemic attack.

^a^ Using Aalen-Johansen estimator accounting for the competing risk of death.

**Figure 1.**
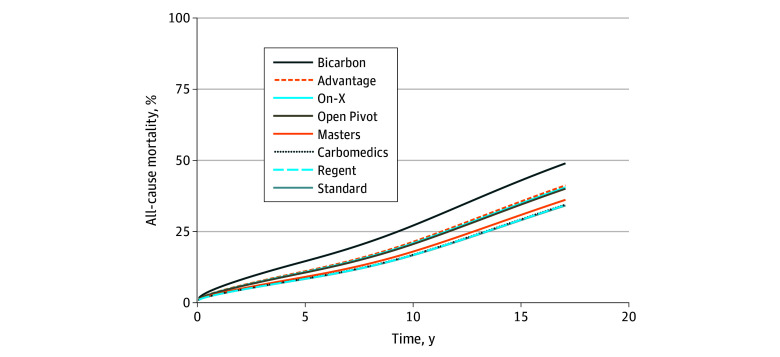
Regression Standardized Cumulative Incidence of All-Cause Mortality After Surgical Aortic Valve Replacement With a Mechanical Valve Prosthesis in Sweden Between 2003 and 2018

**Figure 2.**
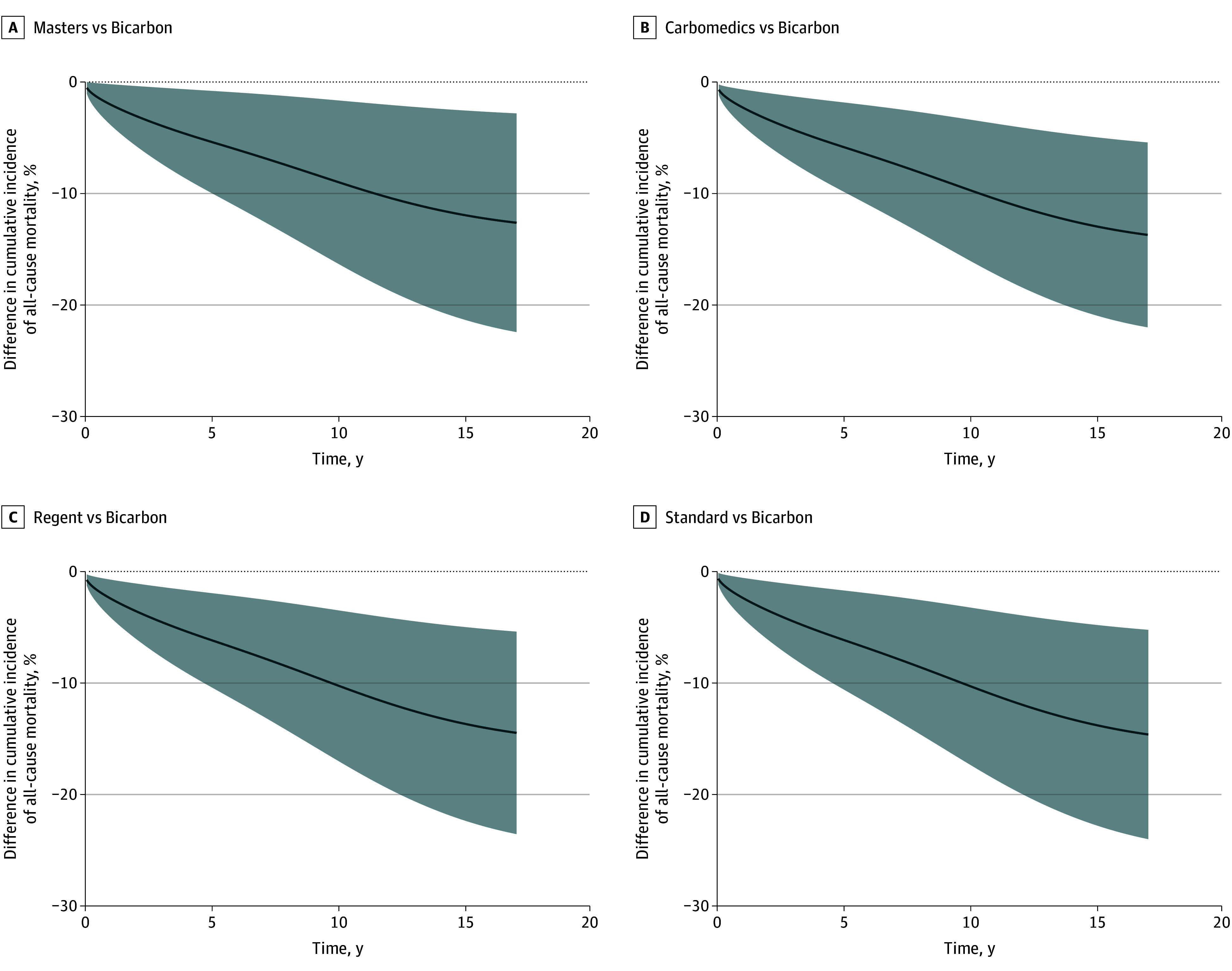
Regression Standardized Difference in Cumulative Incidence of All-Cause Mortality Between the Bicarbon and Other Valve Model Groups After Surgical Aortic Valve Replacement With a Mechanical Valve Prosthesis in Sweden Between 2003 and 2018 Difference in cumulative incidence of all-cause mortality between the Masters model group and Bicarbon model group (A), between the Carbomedics model group and Bicarbon model group (B), between the Regent model group and Bicarbon model group (C), and between the Standard model group and Bicarbon model group (D). The shaded areas indicate 95% CIs. The dashed horizontal lines indicate 0% difference; if the 95% CI crosses this line, the difference is not significant.

After regression standardization, the estimated 10-year all-cause mortality was lower in the Carbomedics model group (17%; 95% CI, 15%-18%), Regent model group (17%; 95% CI, 13%-20%), Standard model group (17%; 95% CI, 14%-19%), and Masters model group (18%; 95% CI, 15%-21%) than in the Bicarbon model group, which had the highest estimated all-cause mortality (27%; 95% CI, 21%-34%) (Figure 1). The regression standardized difference in cumulative incidence in the Bicarbon group vs the Carbomedics, Regent, Standard, and Masters model groups is shown in Figure 2; the mortality differences between the Bicarbon group and the Carbomedics, Regent, Masters, and Standard groups were greater than 10% after 15 years. The regression standardized cumulative incidence data for all-cause mortality are presented in eTable 4 in Supplement 1, and the difference in the cumulative incidence of all-cause mortality between all model groups is shown in eFigure 6 in Supplement 1.

### Reintervention

The total follow-up time for reintervention was 38 245 person-years (mean [SD], 7.3 [4.6] years; maximum, 16.0 years), during which 158 patients (3.0%) underwent an aortic valve reintervention. The crude incidence rate for reintervention per 100 person-years was lowest in the Bicarbon group (0.08; 95% CI, 0.00-0.47) and highest in the Regent group (0.61; 95% CI, 0.38-0.93) (eTable 3 in Supplement 1). The age- and sex-adjusted incidence rate per 100 person-years was lowest in the Bicarbon group (0.09; 95% CI, 0.07-0.11) and highest in the Regent group (0.62; 95% CI, 0.42-0.92). The crude cumulative incidence of reintervention at 10 years was lowest in the Bicarbon model group (0.6%; 95% CI, 0.0%-1.8%) and was highest in the Regent group (5.5%; 95% CI, 3.0%-8.0%) (Table 2). The crude cumulative incidence and the number of events at 5, 10, and 15 years are presented in Table 2. After regression standardization, the estimated cumulative incidence of reintervention at 10 years was lowest in the Bicarbon model group (0.8%; 95% CI, 0.1%-5.5%) and highest in the Masters model group (5.4%; 95% CI, 3.1%-9.1%) (eTable 4 in Supplement 1). There was a statistically significant difference between the Bicarbon and Open Pivot model groups, which both performed better than the Standard, Regent, On-X, and Masters model groups. The regression standardized cumulative incidences of reintervention at 5, 10, and 15 years are presented in eTable 4 in Supplement 1. The regression standardized differences in the cumulative incidence of aortic valve reintervention are shown in eFigure 7 in Supplement 1.

### Heart Failure Hospitalization

The total follow-up time for heart failure hospitalization was 37 678 person-years (mean [SD], 7.2 [4.6] years; maximum, 16.0 years), during which 377 patients (7.2%) were hospitalized for heart failure. The crude incidence rate for heart failure hospitalization per 100 person-years was lowest in the On-X group (0.67; 95% CI, 0.43-1.00) and highest in the Standard group (1.58; 95% CI, 1.26-1.96) (eTable 3 in Supplement 1). Age- and sex-adjusted incidence rates per 100 person-years were lowest in the On-X group (0.76; 95% CI, 0.54-1.08) and the Advantage group (0.76; 95% CI, 0.43-1.36) and highest in the Bicarbon group (1.07; 95% CI, 0.90-1.27).

The crude cumulative incidence for heart failure hospitalization at 10 years was lowest in the On-X model group (6.4%; 95 CI, 3.5%-9.4%) and highest in the Standard group (15.3%; 95% CI, 11.9%-18.8%) (Table 2). The crude cumulative incidence and the number of events at 5, 10 and 15 years are shown in Table 2. After regression standardization, there was no statistically significant difference in the cumulative incidence of 10-year heart failure hospitalization. The regression standardized cumulative incidence was lowest in the Carbomedics model group (6.4%; 95% CI, 5.3%-7.6%) and highest in the Masters model group (9.2%; 95% CI, 6.8%-12.5%) (eTable 4 in Supplement 1). The regression standardized differences in the cumulative incidence of heart failure hospitalization are shown in eFigure 8 in Supplement 1.

### Bleeding Events, Stroke, TIA, or Embolic Events and Sensitivity Analyses

Results regarding bleeding events, stroke, TIA, or embolic events are presented in the eResults and eFigures 9 and 10 in Supplement 1. The results of sensitivity analyses are presented in the eResults and eFigures 11, 12, 13, 14, and 15 in Supplement 1.

### Missing Data

The following variables had missing data: body mass index (407 of 5224 [7.8%]), estimated glomerular filtration rate (124 of 5224 [2.4%]), valve size (77 of 5224 [1.5%]), left ventricular ejection fraction (40 of 5224 [0.8%]), emergency operation (39 of 5224 [0.7%]), and educational status (29 of 5224 [0.6%]).

## Discussion

In this large, population-based, nationwide cohort study, patients who underwent SAVR with the Bicarbon valve prosthesis had a higher mortality risk than patients who underwent SAVR with the Carbomedics, Regent, Masters, or Standard valve prostheses. Patients who underwent SAVR with a Standard, Regent, On-X, or Masters prosthetic valve had a higher risk of aortic valve reintervention than patients who received the Bicarbon or Open Pivot valve prostheses. Patients with a Carbomedics or On-X valve prosthesis had a higher risk of stroke, TIA, or embolic events than patients who received the Regent valve prosthesis.

### Comparison of Multiple Valve Prosthesis Models

In the literature, prosthetic aortic valve model performance is typically studied either within a single valve model series or through direct comparison between 2 valve models.^26,27^ However, multiple valve models can be compared simultaneously using data from national registers, as suggested by Hickey et al,^8^ allowing researchers to identify differences in performance among several valve models. Although these differences may be small in magnitude, they can still be of clinical significance. Swedish national health data registers enabled us to comprehensively assess the performance of all common mechanical aortic valve model prostheses within the same population, with long-term and complete or near-complete follow-up data. Within the Total Population Register, the primary outcome of mortality is tracked both nationally and internationally, ensuring complete follow-up.^12^ Regarding the secondary outcomes of reintervention; heart failure hospitalization; major bleeding events; and stroke, TIA, or embolic events, the only potential for loss to follow-up would arise if patients were to emigrate after undergoing cardiac surgery in Sweden, subsequently seeking further medical care abroad. Given Sweden’s universal health care coverage, we consider this likelihood to be minimal, rendering the follow-up data nearly complete. Thus, the robust data sets that can be obtained from Swedish registers present a unique opportunity to explore the concept of postmarket surveillance of prosthetic valve model groups within a Swedish cohort.

### Valve Model Group Differences

Previous research compared bioprosthetic aortic valve model groups and observed notable differences in performance across these groups.^9^ However, in the present study, when contrasted with the findings of Hickey et al^8^ and Persson et al,^9^ the performance of mechanical aortic valves was largely consistent across the model groups, except for the Bicarbon model group with respect to all-cause mortality. In fact, the estimated differences in heart failure hospitalization and bleeding events were close to 0, indicating virtually no difference at all (eFigures 8 and 9 in Supplement 1).

The Bicarbon model group exhibited a significantly higher all-cause mortality rate than the Carbomedics, Regent, Masters, and Standard model groups. Moreover, given that the mortality differences between the Bicarbon group and the Carbomedics, Regent, Masters, and Standard groups were greater than 10% after 15 years (Figure 2), we interpret this result as reflecting a clinically significant difference in the performance of the Bicarbon valve.

Although existing research on the long-term clinical performance of the Bicarbon valve in direct comparisons with other valves is sparse, studies focusing solely on the Bicarbon valve have generally reported favorable long-term outcomes, including low rates of valve-related complications.^26,28,29,30,31^ For example, Celiento et al^26^ revealed a 17-year survival rate of 47.9% for the Bicarbon valve, along with a 1.9% reintervention rate over the same time period. Furthermore, a study examining hemodynamic performance differences among various valve models concluded that both the Bicarbon and the St Jude Hydrodynamic Plus valves outperformed the Carbomedics valve.^32^ Specifically, the Carbomedics valve exhibited higher gradients, along with lower effective orifice areas and performance indices at 60 days after surgery. Another in vitro study made similar observations, showing that the Bicarbon and St Jude Medical valves had superior hemodynamic profiles in terms of lower shear stress when compared with the Carbomedics and Edwards Duromedics valves.^33^ The superior hemodynamic profile of the Bicarbon valve does not appear to translate into better clinical outcomes. In the study by Hickey et al,^8^ no significant differences were observed in terms of mortality or reintervention rates when comparing the Bicarbon valve with other valve models. The only standout finding in the mechanical valve category in their study pertained to the Medtronic Hall model group, which initially showed a larger unadjusted hazard; however, this difference was negated after statistical adjustments.

Previous studies have generally reported favorable long-term outcomes with the Carbomedics valve.^34,35,36^ Similarly, the St Jude Medical valves are generally considered to be highly reliable.^37^ Comparative analyses between the St Jude Medical and Carbomedics valves have been conducted in several studies, which have suggested comparable short- and long-term mortality rates between the 2 groups.^27,38,39,40,41,42,43^ This similarity has also been confirmed in the present study.

Regarding reintervention, we found that the Bicarbon and Open Pivot model groups outperformed the Standard, Regent, On-X, and Masters model groups. However, given the small number of events in these groups (1 in the Bicarbon group and 2 in the Open Pivot group), these results must be interpreted with caution. Similarly, the observed disparities in the outcome of stroke, TIA, or embolic events may be spurious. Among these groups, the Carbomedics and On-X model valves exhibited poorer performance than the Regent model valves.

### Limitations

This study has some limitations. One is the possibility of residual confounding, which is inherent in cohort studies. Another limitation is the fact that some data were missing. However, the amount of missing data was small, and there was no sign of systematic missingness, meaning that missing data were unlikely to have influenced the results. Another limitation is that the number of patients was small in the Advantage, Bicarbon, and Open Pivot valve model groups, with 167, 164, and 231 patients in each group, respectively. Therefore, the results for these groups should be interpreted with caution. Swedish national health data registries do not contain echocardiography data, which poses a limitation to the types of analyses that we were able to perform.

## Conclusions

In this cohort study, patients who underwent SAVR with the Bicarbon valve prosthesis had a higher rate of all-cause mortality than patients who received the Carbomedics, Regent, Masters, or Standard valve prosthesis. These findings warrant further research on the long-term clinical performance of the Bicarbon valves compared with other mechanical valve prostheses.

## Notes

Supplement 1.
eMethods.

eResults.
**eTable 1.** Number of Different Valve Models Within Each Valve Model Group **eTable 2.** *ICD* Codes Used to Identify the Heart Failure, Bleeding, and Stroke, TIA, and Embolic Event Outcomes **eTable 3.** Crude and Age-, and Sex-Adjusted Incidence Rates per 100 Person-Years (95% CI) for All-Cause Mortality, Reintervention, Heart Failure Hospitalization, Bleeding Event, and Stroke, TIA, or Embolic Event Following SAVR With a Mechanical Valve Prosthesis in Sweden Between 2003 and 2018 **eTable 4.** Regression Standardized Cumulative Incidence for All-Cause Mortality, Reintervention, Heart Failure Hospitalization, Bleeding and Stroke, TIA or Embolic Event Following Surgical Aortic Valve Replacement With a Mechanical Valve Prosthesis in Sweden Between 2003 and 2018 % (95% CI) **eFigure 1.** Number of Implanted Mechanical Valve Prostheses per Model Group and Year in Sweden Between 2003 and 2018 **eFigure 2.** Age Distribution per Model Group After Surgical Aortic Valve Replacement With a Mechanical Valve Prosthesis in Sweden Between 2003 and 2018 **eFigure 3.** Proportion of Prosthesis Valve Size per Model Group Following Surgical Aortic Valve Replacement With a Mechanical Valve Prosthesis in Sweden Between 2003 and 2018 **eFigure 4.** Proportion of Left Ventricular Ejection Fraction at Surgery per Model Group Following Surgical Aortic Valve Replacement With a Mechanical Valve Prosthesis in Sweden Between 2003 and 2018 **eFigure 5.** Proportion of Patient Sex per Model Group Following Surgical Aortic Valve Replacement With a Mechanical Valve Prosthesis in Sweden Between 2003 and 2018 **eFigure 6.** The Regression Standardized Difference in Cumulative Incidence of All-Cause Mortality Following Surgical Aortic Valve Replacement With a Mechanical Valve Prosthesis in Sweden Between 2003 and 2018 **eFigure 7.** The Regression Standardized Difference in Cumulative Incidence of Aortic Valve Reintervention Following Surgical Aortic Valve Replacement With a Mechanical Valve Prosthesis in Sweden Between 2003 and 2018 **eFigure 8.** The Regression Standardized Difference in Cumulative Incidence of Heart Failure Hospitalization Following Surgical Aortic Valve Replacement With a Mechanical Valve Prosthesis in Sweden Between 2003 and 2018 **eFigure 9.** The Regression Standardized Difference in Cumulative Incidence of Bleeding Events Following Surgical Aortic Valve Replacement With a Mechanical Valve Prosthesis in Sweden Between 2003 and 2018 **eFigure 10.** The Regression Standardized Difference in Cumulative Incidence of Stroke, TIA or Embolic Event Following Surgical Aortic Valve Replacement With a Mechanical Valve Prosthesis in Sweden Between 2003 and 2018 **eFigure 11.** Kaplan-Meier Estimated Survival and Number at Risk After Surgical Aortic Valve Replacement With Different Mechanical Valve Model Groups in Sweden Between 2003 and 2018 **eFigure 12.** Aalen-Johansen Estimated Aortic Valve Reintervention and Number at Risk After Surgical Aortic Valve Replacement With Different Mechanical Valve Model Groups in Sweden Between 2003 and 2018 **eFigure 13.** Aalen-Johansen Estimated Heart Failure Hospitalization and Number at Risk After Surgical Aortic Valve Replacement With Different Mechanical Valve Model Groups in Sweden Between 2003 and 2018 **eFigure 14.** Aalen-Johansen Estimated Major Bleeding Event and Number at Risk After Surgical Aortic Valve Replacement With Different Mechanical Valve Model Groups in Sweden Between 2003 and 2018 **eFigure 15.** Aalen-Johansen Estimated Stroke, TIA or Embolic Event and Number at Risk After Surgical Aortic Valve Replacement With Different Mechanical Valve Model Groups in Sweden Between 2003 and 2018

Supplement 2.
Data Sharing Statement
