# Supplementary material for: Comparative Long-Term Clinical Performance of Mechanical Aortic Valve Prostheses
Source: JAMA Netw Open. 2024 Apr 19;7(4):e247525. doi: 10.1001/jamanetworkopen.2024.7525 (PMC11031681; doi:10.1001/jamanetworkopen.2024.7525)
Supplement: Supplement 1. — eMethods. eResults. eTable 1. Number of Different Valve Models Within Each Valve Model Group eTable 2. ICD Codes Used to Identify the Heart Failure, Bleeding, and Stroke, TIA, and Embolic Event Outcomes eTable 3. Crude and Age-, and Sex-Adjusted Incidence Rates per 100 Person-Years (95% CI) for All-Cause Mortality, Reintervention, Heart Failure Hospitalization, Bleeding Event, and Stroke, TIA, or Embolic Event Following SAVR With a Mechanical Valve Prosthesis in Sweden Between 2003 and 2018 eTable 4. Regression Standardized Cumulative Incidence for All-Cause Mortality, Reintervention, Heart Failure Hospitalization, Bleeding and Stroke, TIA or Embolic Event Following Surgical Aortic Valve Replacement With a Mechanical Valve Prosthesis in Sweden Between 2003 and 2018 % (95% CI) eFigure 1. Number of Implanted Mechanical Valve Prostheses per Model Group and Year in Sweden Between 2003 and 2018 eFigure 2. Age Distribution per Model Group After Surgical Aortic Valve Replacement With a Mechanical Valve Prosthesis in Sweden Between 2003 and 2018 eFigure 3. Proportion of Prosthesis Valve Size per Model Group Following Surgical Aortic Valve Replacement With a Mechanical Valve Prosthesis in Sweden Between 2003 and 2018 eFigure 4. Proportion of Left Ventricular Ejection Fraction at Surgery per Model Group Following Surgical Aortic Valve Replacement With a Mechanical Valve Prosthesis in Sweden Between 2003 and 2018 eFigure 5. Proportion of Patient Sex per Model Group Following Surgical Aortic Valve Replacement With a Mechanical Valve Prosthesis in Sweden Between 2003 and 2018 eFigure 6. The Regression Standardized Difference in Cumulative Incidence of All-Cause Mortality Following Surgical Aortic Valve Replacement With a Mechanical Valve Prosthesis in Sweden Between 2003 and 2018 eFigure 7. The Regression Standardized Difference in Cumulative Incidence of Aortic Valve Reintervention Following Surgical Aortic Valve Replacement With a Mechanical Valve Prosthesis in Sweden Between [file jamanetwopen-e247525-s001.pdf]

Supplementary Online Content

Granbom Koski M, Glaser N, Franco-Cereceda A, Sartipy U, Dismorr M. Comparative long-term clinical performance of mechanical aortic valve prostheses. *JAMA Netw Open.* 2024;7(4):e247525. doi:10.1001/jamanetworkopen.2024.7525

eMethods.
eResults.
eTable 1. Number of Different Valve Models Within Each Valve Model Group
eTable 2. *ICD* Codes Used to Identify the Heart Failure, Bleeding, and Stroke, TIA, and Embolic Event Outcomes
eTable 3. Crude and Age-, and Sex-Adjusted Incidence Rates per 100 Person-Years (95% CI) for All-Cause Mortality, Reintervention, Heart Failure Hospitalization, Bleeding Event, and Stroke, TIA, or Embolic Event Following SAVR With a Mechanical Valve Prosthesis in Sweden Between 2003 and 2018
eTable 4. Regression Standardized Cumulative Incidence for All-Cause Mortality, Reintervention, Heart Failure Hospitalization, Bleeding and Stroke, TIA or Embolic Event Following Surgical Aortic Valve Replacement With a Mechanical Valve Prosthesis in Sweden Between 2003 and 2018 % (95% CI)
eFigure 1. Number of Implanted Mechanical Valve Prostheses per Model Group and Year in Sweden Between 2003 and 2018
eFigure 2. Age Distribution per Model Group After Surgical Aortic Valve Replacement With a Mechanical Valve Prosthesis in Sweden Between 2003 and 2018
eFigure 3. Proportion of Prosthesis Valve Size per Model Group Following Surgical Aortic Valve Replacement With a Mechanical Valve Prosthesis in Sweden Between 2003 and 2018
eFigure 4. Proportion of Left Ventricular Ejection Fraction at Surgery per Model Group Following Surgical Aortic Valve Replacement With a Mechanical Valve Prosthesis in Sweden Between 2003 and 2018
eFigure 5. Proportion of Patient Sex per Model Group Following Surgical Aortic Valve Replacement With a Mechanical Valve Prosthesis in Sweden Between 2003 and 2018

© 2024 Granbom Koski M et al. *JAMA Network Open.*

eFigure 6. The Regression Standardized Difference in Cumulative Incidence of All-Cause Mortality Following Surgical Aortic Valve Replacement With a Mechanical Valve Prosthesis in Sweden Between 2003 and 2018

eFigure 7. The Regression Standardized Difference in Cumulative Incidence of Aortic Valve Reintervention Following Surgical Aortic Valve Replacement With a Mechanical Valve Prosthesis in Sweden Between 2003 and 2018

eFigure 8. The Regression Standardized Difference in Cumulative Incidence of Heart Failure Hospitalization Following Surgical Aortic Valve Replacement With a Mechanical Valve Prosthesis in Sweden Between 2003 and 2018

eFigure 9. The Regression Standardized Difference in Cumulative Incidence of Bleeding Events Following Surgical Aortic Valve Replacement With a Mechanical Valve Prosthesis in Sweden Between 2003 and 2018

eFigure 10. The Regression Standardized Difference in Cumulative Incidence of Stroke, TIA or Embolic Event Following Surgical Aortic Valve Replacement With a Mechanical Valve Prosthesis in Sweden Between 2003 and 2018

eFigure 11. Kaplan-Meier Estimated Survival and Number at Risk After Surgical Aortic Valve Replacement With Different Mechanical Valve Model Groups in Sweden Between 2003 and 2018

eFigure 12. Aalen-Johansen Estimated Aortic Valve Reintervention and Number at Risk After Surgical Aortic Valve Replacement With Different Mechanical Valve Model Groups in Sweden Between 2003 and 2018

eFigure 13. Aalen-Johansen Estimated Heart Failure Hospitalization and Number at Risk After Surgical Aortic Valve Replacement With Different Mechanical Valve Model Groups in Sweden Between 2003 and 2018

eFigure 14. Aalen-Johansen Estimated Major Bleeding Event and Number at Risk After Surgical Aortic Valve Replacement With Different Mechanical Valve Model Groups in Sweden Between 2003 and 2018

eFigure 15. Aalen-Johansen Estimated Stroke, TIA or Embolic Event and Number at Risk After Surgical Aortic Valve Replacement With Different Mechanical Valve Model Groups in Sweden Between 2003 and 2018

This supplementary material has been provided by the authors to give readers additional information about their work.

© 2024 Granbom Koski M et al. *JAMA Network Open.*

eMethods.

Missing data - Classification and regression tree imputation

The Classification and regression tree (CART) algorithm was developed in 1984 by Breiman and colleagues and has become a popular class of machine learning. It can be used to classify, or predict, variables depending on other factors. In the case of a discrete variable of interest, a classical classification tree identifies the outcome group based on Gini impurity. In the case of a continuous variable of interest, the algorithm utilizes a regression model to estimate the variable and perform splits in the tree based on minimizing the residual sum of squares.

The advantages of using the CART algorithm in imputation is that it can handle binary, categorical, and continuous variables both as predictors and as target variables. It is also robust against outliers. It has been implemented in the R package *rpart* which is used as a computational backend in the package *simputation* for missing imputation with CART. It is also implemented in the *mice* package as one of the available imputation methods to be used with multiple imputation by chained equations. For further reading we recommend Stef van Buuren, Flexible imputation of missing data, Second edition, Chapman & Hall/CRC, 2021.

Survival model

The following variables were included in the final survival model: Model group, age, sex, hospital, left ventricular ejection fraction, emergent status, concomitant coronary artery bypass grafting, atrial fibrillation, alcohol dependency, preoperative cancer, prior chronic obstructive pulmonary disease, prior diabetes, prior heart failure, hypertension, hepatic disease, concomitant ascending aortic surgery, prior stroke, pacemaker or implantable cardioverter device, disposable family income, BMI, period of surgery, estimated glomerular filtration rate, and valve size. The baseline hazard was modelled as a natural spline with three degrees of freedom.

Aortic reintervention model

The following variables were included in the final reintervention model: Model group, age, sex, hospital, left ventricular ejection fraction, emergent status, concomitant ascending aortic surgery, birth region, diabetes, peripheral vascular disease, prior

© 2024 Granbom Koski M et al. *JAMA Network Open*.

percutaneous coronary intervention, estimated glomerular filtration rate, and valve size. The baseline hazard was modelled as a 3rd degree exponential B-spline.

Heart failure hospitalization model

The following variables were included in the final heart failure model: Model group, age, sex, hospital, left ventricular ejection fraction, concomitant coronary artery bypass grafting, prior atrial fibrillation, prior myocardial infarction, diabetes, prior heart failure, hepatic disease, prior percutaneous coronary intervention, disposable family income, body mass index, estimated glomerular filtration rate, and valve size. The baseline hazard was modelled as a 3rd degree exponential B-spline.

Bleeding event model

The following variables were included in the final bleeding event model: Model group, age, sex, hospital, emergent status, concomitant coronary artery bypass grafting, concomitant ascending aortic surgery, alcohol dependency, prior myocardial infarction, chronic obstructive pulmonary disease, prior endocarditis, prior heart failure, hypertension, hepatic disease, prior percutaneous coronary intervention, disposable family income, period of surgery, estimated glomerular filtration rate, and valve size. The baseline hazard was modelled as a 3rd degree exponential B-spline.

Stroke, TIA , or embolic event model

The following variables were included in the final stroke, TIA, or embolic event model: Model group, age, sex, hospital, emergent status, concomitant coronary artery bypass grafting, concomitant ascending aortic surgery, diabetes, hyperlipidemia, hepatic disease, prior stroke, period of surgery, and valve size. The baseline hazard was modelled as a 3rd degree exponential B-spline.

© 2024 Granbom Koski M et al. *JAMA Network Open*.

eResults

Bleeding events

The total follow-up time for a bleeding event was 36891 person-years (mean 7.1 years, maximum 16.0 years), during which 458 patients (9%) had a bleeding event. The crude incidence rate for bleeding events was the lowest in the Bicarbon group (0.88 per 100 person-years, 95% CI 0.42-1.62), and the highest in the Carbomedics group (1.15 per 100 person-years, 95% CI 1.00-1.31). The age- and sex-adjusted incidence rate per 100 person-years was lowest in the Bicarbon model group (0.9 per 100 person-years, 95% CI 0.8-1.0). The crude and age-, and sex-adjusted incidence rates per 100 person-years are shown in eTable 3. The crude cumulative incidence for bleeding events was the lowest in the Bicarbon group at 10 years (7.5%, 95% CI 2.5-12.5%), and highest in the Standard model group at 10 years (11.7%, 95% CI 8.6-14.8%). The crude cumulative incidence and number of events at 5, 10, and 15 years is presented in Table 2.

After regression standardization, there was no statistically significant difference in the estimated cumulative incidence of bleeding events at 10 years. The estimated cumulative incidence of bleeding events at 10 years was the lowest in the Standard group (7.2%, 95% CI 5.2-10.1%), and highest in the On-X group (10.1%, 95% CI 7.2-14.0%). The regression standardized cumulative incidence of bleeding events are presented in eTable 4. The regression standardized cumulative incidence differences in bleeding events are shown in eFigure 9.

Stroke, TIA, or embolic event

The total follow-up time for an event of stroke, TIA or an embolic event was 37010 person-years (mean 7.1 years, maximum 16.0 years), during which 433 patients (8%) experienced an event. The crude incidence rate per 100 person-years for stroke, TIA, or embolic event was the lowest in the Regent group (0.85, 95% CI 0.57-1.21) and the Masters group (0.85, 95% CI 0.64-1.09). The crude incidence rate per 100 person-years was highest in the On-X group (1.24, 95% CI 0.89-1.66). The age- and sex-adjusted incidence rates per 100 person-years was highest in the On-X group (1.4, 95% CI 1.0-2.0) and lowest in the Standard (0.8, 95% CI 0.4-1.3), the Regent (0.8, 95% CI 0.6-1.1) and the Masters model groups (0.8, 95% CI 0.6-1.3). The crude and age-, and sex-adjusted incidence rates for stroke, TIA, or embolic events per 100 person-years are

© 2024 Granbom Koski M et al. *JAMA Network Open*.

shown in eTable 3. The crude 10-year cumulative incidence for stroke, TIA, or embolic event was lowest in the Open Pivot group (6.4%, 95% CI 2.8-9.9%) and highest in the Standard group (11.5%, 95% CI 8.4-14.5%). The crude cumulative incidence and number of events at 5, 10 and 15 years are presented in Table 2. After regression standardization, the estimated 10-year cumulative incidence of stroke, TIA, or embolic events was lowest in the Regent group (6.2%, 95% CI 4.2-9.1%), and highest in the Carbomedics group (10.6%, 95% CI 8.8-12.7%). There was a statistically significant difference between the Regent model group and the Carbomedics and On-X model groups. The regression standardized cumulative incidence is presented in eTable 4. The regression standardized cumulative incidence differences in stroke, TIA, or embolic events are shown in eFigure 10.

Sensitivity analyses

We repeated the main analyses using complete cases, and in the isolated aortic valve replacement subgroup, and excluding the Top Hat model with similar results. As a sensitivity analysis, we analyzed survival difference in the Bicarbon and Regent model groups using inverse probability of treatment weighting utilizing optimization-based weights. The survival benefit of the Regent valve compared to Bicarbon was coherent to the main analysis using this method (HR 0.58, 95% CI 039-0.86).

© 2024 Granbom Koski M et al. *JAMA Network Open.*

eTable 1. Number of Different Valve Models Within Each Valve Model Group

	No.
On-X	
On-X Aortic	362
On-X Aortic Conform-X Sewing Cuff	85
On-X Aortic SERIALNO	5
On-X Aortic Valve Sewing Ring	227
Carbomedics	
Carbomedics Carbo-Seal AP	298
Carbomedics Carbo-Seal Valsalva CP	131
Carbomedics Reduced Aortic R5	1,375
Carbomedics SERIALNO	228
Carbomedics Standard Aortic A5	28
Carbomedics Top Hat Supraannular Aortic S5	105
Carbomedics Unknown	6
Bicarbon	
Bicarbon Aortic LN	16
Bicarbon Overline Aortic LOV	1
Bicarbon Slimline Aortic LSA	147
Standard	
St. Jude Composite Aortic Valved Graft Unknown	39
St. Jude Mechanical Aortic Standard A-101	77
St. Jude SERIALNO	302
Regent	
Regent aortic MHV AGN-751	411
Regent aortic MHV flex cuff AGFN-756	156
Open Pivot	
ATS Open Pivot Aortic Valved Graft (AVG) 502AG	12
ATS Open Pivot AP Series Heart Valve 501DA	43

© 2024 Granbom Koski M et al. *JAMA Network Open.*

ATS Open Pivot AP Series Heart Valve 505DA	51
ATS Open Pivot AP series Heart Valve AP360	3
ATS Open Pivot Standard Heart Valve 500DM	1
ATS Open Pivot Standard Heart Valve 500FA	121
Masters	
Aortic HP Masters mechanic heart valve AHPJ-505	443
Aortic Masters Series	185
Masters series CAVG compositegraft CAVGJ-514 00	118
Masters Series MTJ-503	2
Masters Series VAVGJ Composite	61
Masters Standard Aortic mechanic heart valve AJ-501	18
Advantage	
Advantage Aorta A7760	107
Advantage Aorta Supra A7760SA	23
Advantage Aorta Unknown	37

SERIALNO and Unknown indicates only model group is known, but the specific model could not be determined

© 2024 Granbom Koski M et al. *JAMA Network Open*.

eTable 2. *ICD* Codes Used to Identify the Heart Failure, Bleeding, and Stroke, TIA, and Embolic Event Outcomes

Heart failure
I50
Bleeding
I60, I61, I62, I850, K226, K250, K252, K254, K256, K260, K262, K264, K266, K270, K272, K274, K276, K280, K282, K284, K286, K290, K625, K920, K921, K922, D629, D500, R040, R041, R042, R048, R049, R319, I312, J942
Stroke, TIA, and embolic event
I63, I64, G458, G459, I74

© 2024 Granbom Koski M et al. *JAMA Network Open*.

eTable 3. Crude and Age-, and Sex-Adjusted Incidence Rates per 100 Person-Years (95% CI) for All-Cause Mortality, Reintervention, Heart Failure Hospitalization, Bleeding Event, and Stroke, TIA, or Embolic Event Following SAVR With a Mechanical Valve Prosthesis in Sweden Between 2003 and 2018

Model group	All-cause mortality	Reintervention	Heart failure hospitalization	Bleeding event	Stroke, TIA, or embolic event
Crude					
On-X	1.6 (1.2–2.0)	0.47 (0.27–0.76)	0.67 (0.43–1.00)	1.02 (0.72–1.42)	1.24 (0.89–1.66)
Carbomedics	2.3 (2.1–2.5)	0.34 (0.27–0.43)	0.71 (0.60–0.84)	1.15 (1.00–1.31)	1.04 (0.90–1.20)
Bicarbon	3.2 (2.3–4.4)	0.08 (0.00–0.47)	1.07 (0.55–1.87)	0.88 (0.42–1.62)	1.14 (0.61–1.95)
Standard	6.7 (6.0–7.5)	0.24 (0.13–0.41)	1.58 (1.26–1.96)	1.11 (0.85–1.43)	1.14 (0.87–1.47)
Regent	2.0 (1.6–2.5)	0.61 (0.38–0.93)	0.96 (0.66–1.34)	1.07 (0.76–1.47)	0.85 (0.57–1.21)
Open Pivot	2.1 (1.4–2.9)	0.14 (0.02–0.52)	0.74 (0.36–1.37)	1.13 (0.63–1.87)	0.88 (0.46–1.54)
Masters	2.2 (1.9–2.6)	0.39 (0.26–0.57)	0.93 (0.71–1.19)	0.95 (0.73–1.21)	0.85 (0.64–1.09)
Advantage	2.8 (2.1–3.8)	0.35 (0.13–0.77)	0.78 (0.42–1.34)	1.05 (0.61–1.68)	0.91 (0.51–1.49)
Age-, and sex-adjusted					
On-X	2.2 (1.8–2.7)	0.43 (0.28–0.65)	0.76 (0.54–1.08)	1.0 (0.7–1.4)	1.4 (1.0–2.0)
Carbomedics	2.8 (2.1–3.8)	0.35 (0.05–2.47)	0.77 (0.43–1.38)	1.1 (0.6–2.2)	1.1 (0.6–1.8)
Bicarbon	3.5 (3.2–3.9)	0.09 (0.07–0.11)	1.07 (0.90–1.27)	0.9 (0.8–1.0)	1.1 (1.0–1.3)
Standard	3.5 (2.7–4.7)	0.49 (0.21–1.15)	0.96 (0.55–1.67)	1.1 (0.7–1.8)	0.8 (0.4–1.3)
Regent	2.1 (1.8–2.5)	0.62 (0.42–0.92)	0.97 (0.76–1.26)	1.1 (0.8–1.4)	0.8 (0.6–1.1)
Open Pivot	2.3 (1.6–3.5)	0.15 (0.04–0.60)	0.91 (0.44–1.88)	1.1 (0.7–1.9)	0.9 (0.5–1.7)
Masters	2.6 (2.2–3.0)	0.41 (0.23–0.73)	0.95 (0.70–1.30)	1.0 (0.7–1.3)	0.8 (0.6–1.3)
Advantage	3.1 (2.1–4.6)	0.37 (0.22–0.60)	0.76 (0.43–1.36)	1.0 (0.7–1.5)	0.9 (0.7–1.3)

CI = confidence interval

© 2024 Granbom Koski M et al. *JAMA Network Open*.

eTable 4. Regression Standardized Cumulative Incidence for All-Cause Mortality, Reintervention, Heart Failure Hospitalization, Bleeding and Stroke, TIA or Embolic Event Following Surgical Aortic Valve Replacement With a Mechanical Valve Prosthesis in Sweden Between 2003 and 2018 % (95% CI)

	5 years	10 years	15 years
All-cause mortality			
On-X	10.6 (8.2–13.1)	20.9 (16.8–25.0)	35.0 (29.4–40.7)
Carbomedics	8.3 (7.4–9.3)	16.8 (15.3–18.4)	29.4 (27.0–31.8)
Bicarbon	14.5 (10.3–18.7)	27.2 (20.7–33.7)	43.2 (34.9–51.4)
Standard	8.2 (6.8–9.6)	16.6 (14.1–19.1)	29.1 (25.2–33.0)
Regent	8.2 (6.4–10.1)	16.7 (13.5–20.0)	29.2 (24.4–34.0)
Open Pivot	10.4 (7.2–13.7)	20.6 (15.1–26.1)	34.6 (27.0–42.2)
Masters	8.9 (7.2–10.6)	17.9 (15.0–20.9)	30.9 (26.6–35.3)
Advantage	10.9 (8.0–13.9)	21.4 (16.5–26.3)	35.7 (29.0–42.4)
Reintervention			
On-X	2.6 (1.5–4.3)	3.8 (2.2–6.3)	6.3 (3.7–10.6)
Carbomedics	1.6 (1.2–2.1)	2.3 (1.8–3.0)	4.0 (3.0–5.2)
Bicarbon	0.5 (0.1–3.8)	0.8 (0.1–5.5)	1.3 (0.2–9.0)
Standard	3.6 (1.9–6.7)	5.3 (2.8–9.7)	8.9 (4.9–16.1)
Regent	3.1 (1.9–4.9)	4.5 (2.8–7.1)	7.6 (4.7–12.2)
Open Pivot	0.8 (0.2–3.3)	1.2 (0.3–4.8)	2.1 (0.5–8.2)
Masters	3.7 (2.1–6.4)	5.4 (3.1–9.1)	8.9 (5.2–15.1)
Advantage	1.9 (0.8–4.3)	2.7 (1.2–6.2)	4.5 (2.0–10.2)
Heart failure hospitalization			
On-X	4.0 (2.6–6.1)	7.6 (5.1–11.3)	11.5 (7.7–17.0)
Carbomedics	3.2 (2.6–4.0)	6.4 (5.3–7.6)	10.0 (8.4–11.9)
Bicarbon	4.6 (2.6–8.1)	8.5 (4.9–14.6)	12.5 (7.2–21.3)
Standard	3.7 (2.7–5.1)	7.3 (5.4–9.8)	11.4 (8.5–15.3)
Regent	3.5 (2.4–5.1)	6.9 (4.8–9.8)	10.8 (7.5–15.3)
Open Pivot	3.9 (2.2–7.1)	7.6 (4.3–13.3)	11.6 (6.6–20.2)
Masters	4.9 (3.5–6.7)	9.2 (6.8–12.5)	14.1 (10.3–19.0)
Advantage	3.5 (2.0–6.0)	6.7 (4.0–11.2)	10.1 (6.0–16.7)

© 2024 Granbom Koski M et al. *JAMA Network Open*.

Bleeding event			
On-X	6.1 (4.3–8.6)	10.1 (7.2–14.0)	14.1 (10.1–19.5)
Carbomedics	5.7 (4.8–6.8)	9.6 (8.1–11.3)	13.8 (11.7–16.4)
Bicarbon	5.1 (2.7–9.4)	8.2 (4.4–15.1)	11.3 (6.1–20.5)
Standard	4.3 (3.1–6.0)	7.3 (5.2–10.1)	10.6 (7.5–14.7)
Regent	5.5 (3.9–7.9)	9.3 (6.6–13.0)	13.3 (9.4–18.6)
Open Pivot	5.7 (3.4–9.3)	9.4 (5.7–15.1)	13.1 (8.0–20.9)
Masters	5.2 (3.8–7.2)	8.7 (6.4–11.9)	12.4 (9.0–17.0)
Advantage	5.5 (3.4–9.0)	9.2 (5.7–14.6)	12.8 (8.0–20.3)
Stroke, TIA or embolic event			
On-X	5.9 (4.3–8.2)	10.5 (7.7–14.3)	15.0 (11.0–20.4)
Carbomedics	5.8 (4.8–7.1)	10.6 (8.8–12.7)	15.6 (12.9–18.7)
Bicarbon	4.7 (2.6–8.3)	8.2 (4.7–14.3)	11.6 (6.6–19.9)
Standard	5.4 (3.7–7.7)	9.7 (6.8–13.9)	14.3 (10.0–20.2)
Regent	3.4 (2.3–5.0)	6.2 (4.2–9.1)	9.2 (6.3–13.5)
Open Pivot	3.9 (2.2–6.9)	7.1 (4.1–12.2)	10.2 (5.9–17.4)
Masters	4.6 (3.3–6.5)	8.4 (6.0–11.7)	12.2 (8.7–16.9)
Advantage	5.4 (3.2–9.1)	9.6 (5.7–15.8)	13.7 (8.3–22.4)
CI = confidence interval			

© 2024 Granbom Koski M et al. *JAMA Network Open*.

eFigure 1. Number of Implanted Mechanical Valve Prostheses per Model Group and Year in Sweden Between 2003 and 2018

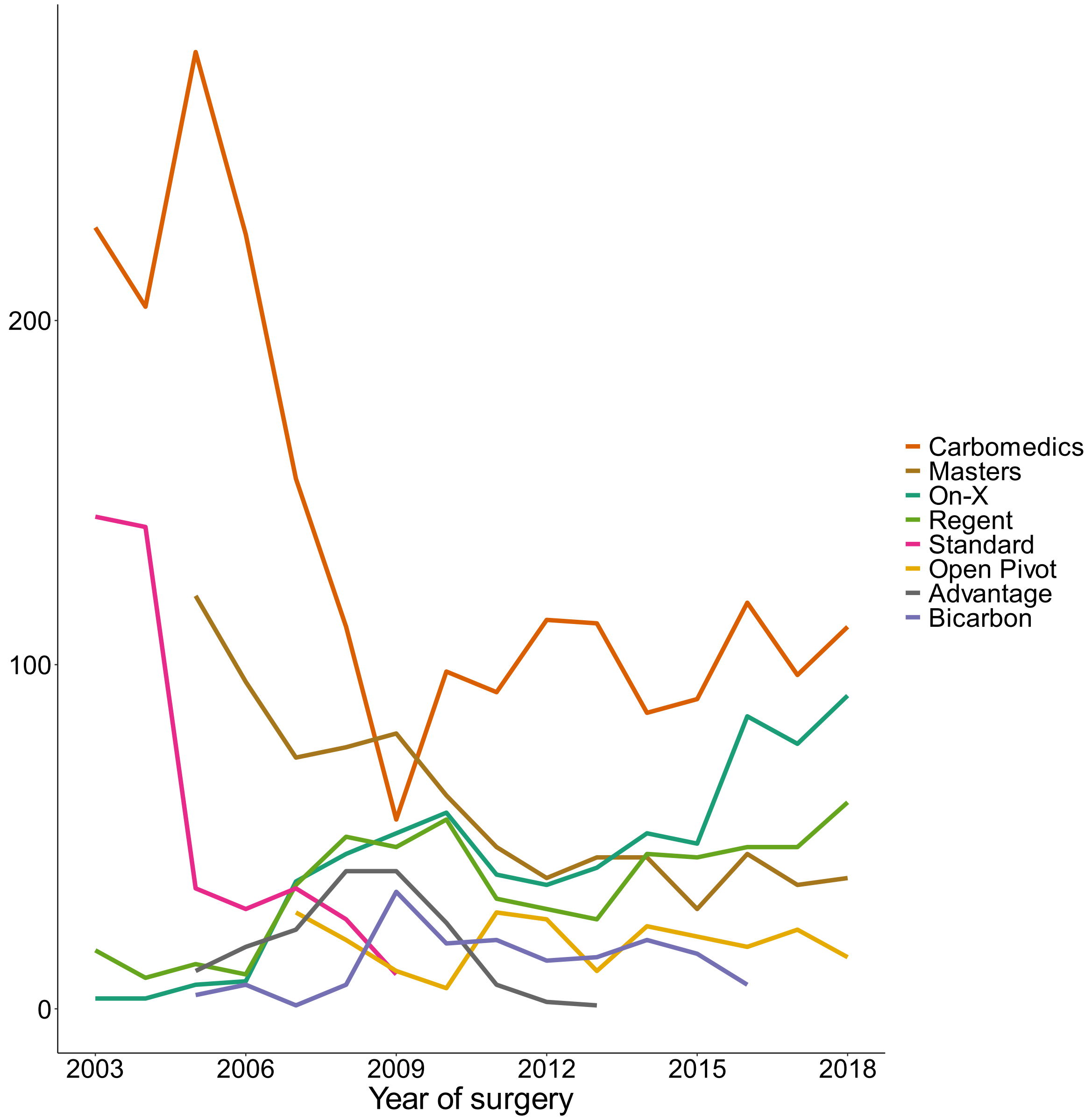

© 2024 Granbom Koski M et al. *JAMA Network Open*.

eFigure 2. Age Distribution per Model Group After Surgical Aortic Valve Replacement With a Mechanical Valve Prosthesis in Sweden Between 2003 and 2018

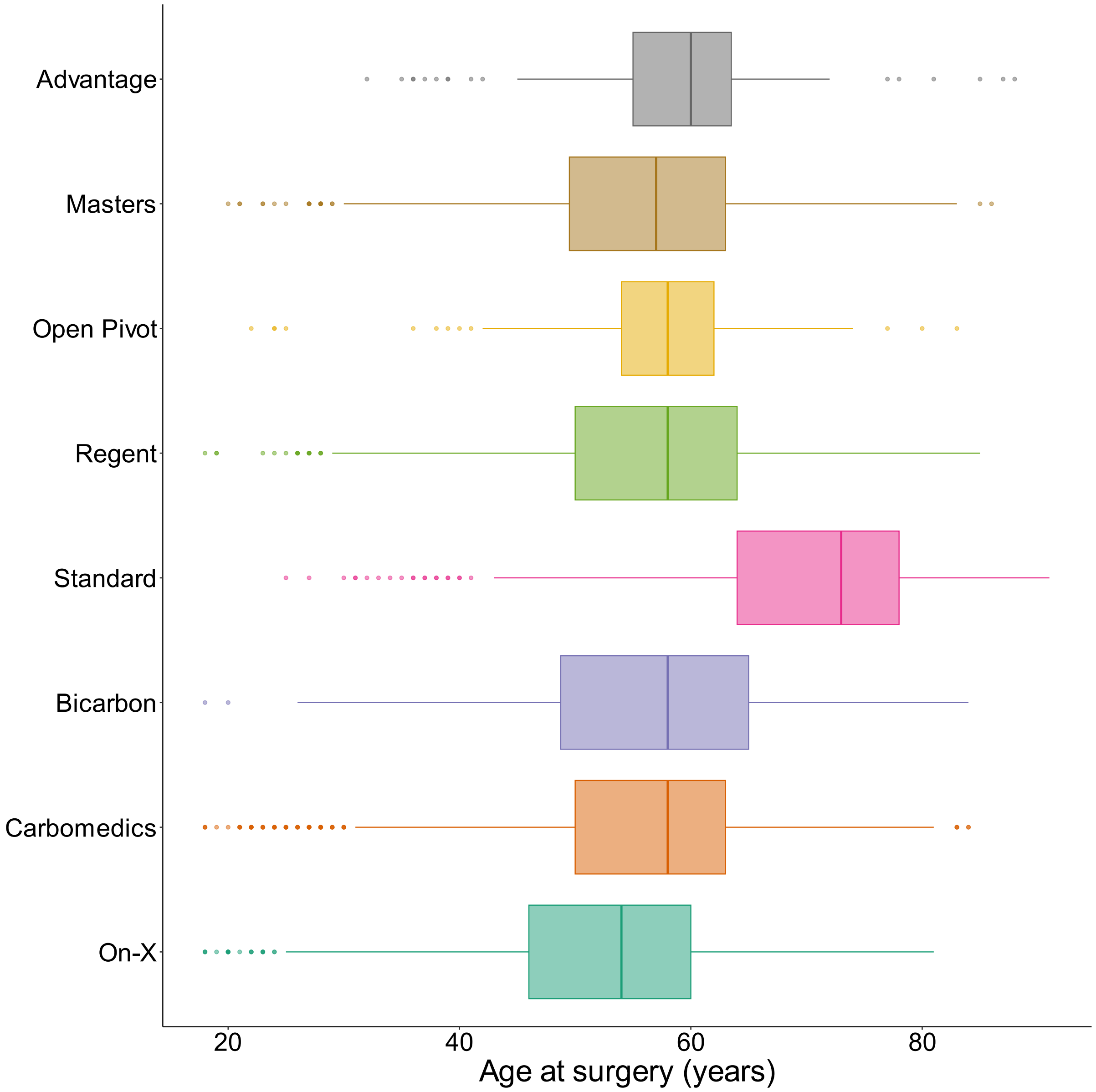

© 2024 Granbom Koski M et al. *JAMA Network Open*.

eFigure 3. Proportion of Prosthesis Valve Size per Model Group Following Surgical Aortic Valve Replacement With a Mechanical Valve Prosthesis in Sweden Between 2003 and 2018

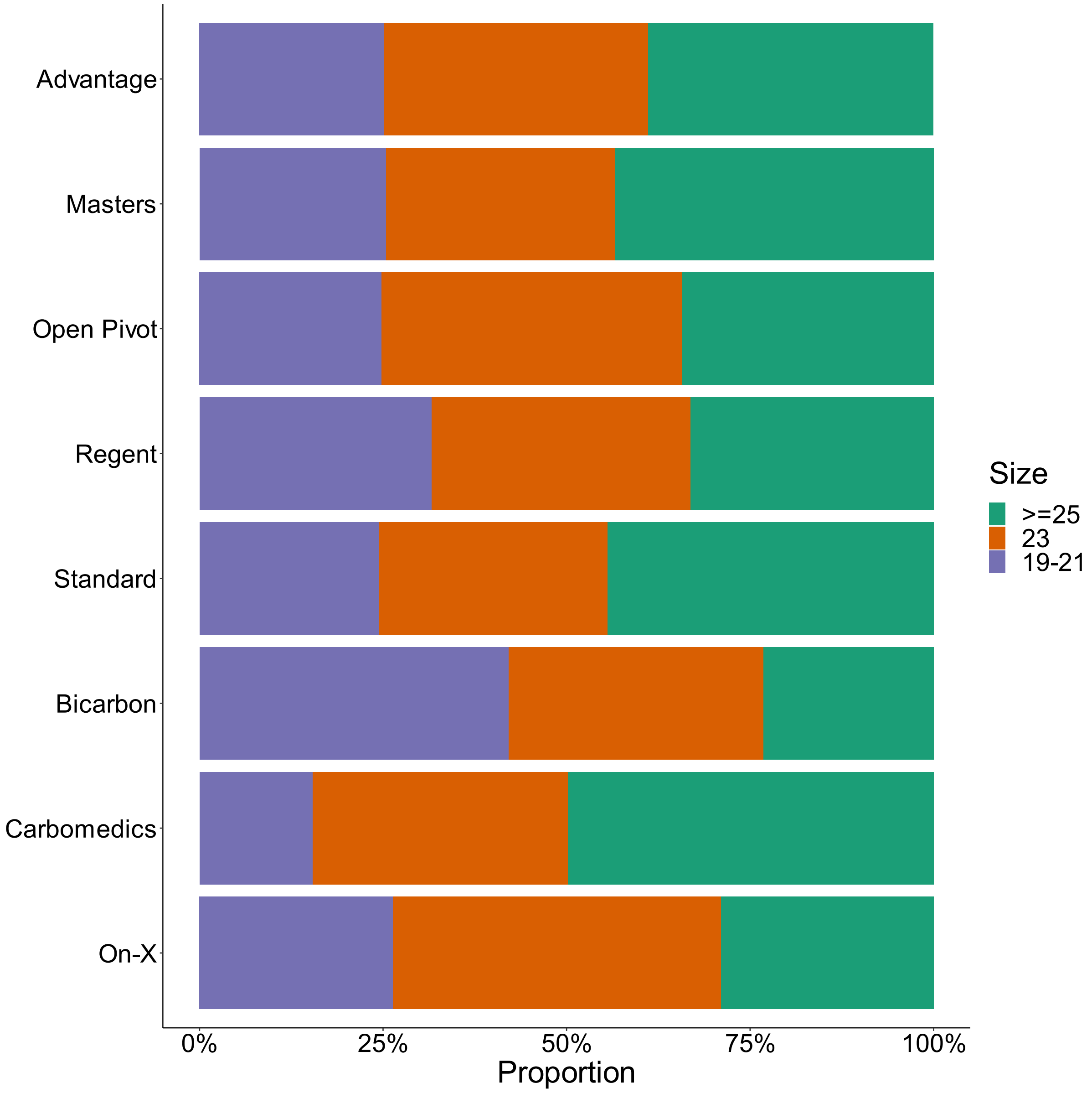

© 2024 Granbom Koski M et al. *JAMA Network Open*.

eFigure 4. Proportion of Left Ventricular Ejection Fraction at Surgery per Model Group Following Surgical Aortic Valve Replacement With a Mechanical Valve Prosthesis in Sweden Between 2003 and 2018

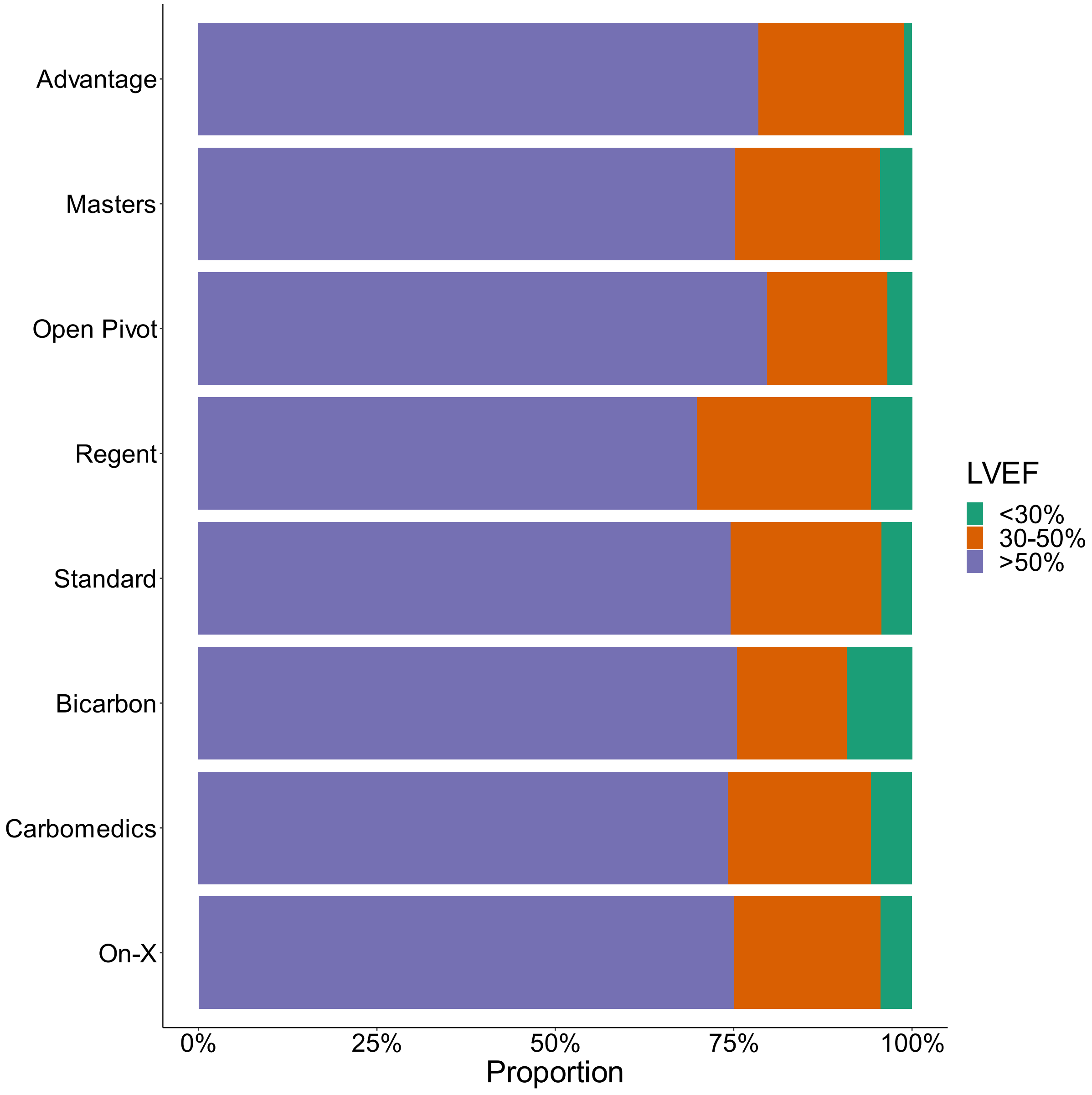

© 2024 Granbom Koski M et al. *JAMA Network Open*.

eFigure 5. Proportion of Patient Sex per Model Group Following Surgical Aortic Valve Replacement With a Mechanical Valve Prosthesis in Sweden Between 2003 and 2018

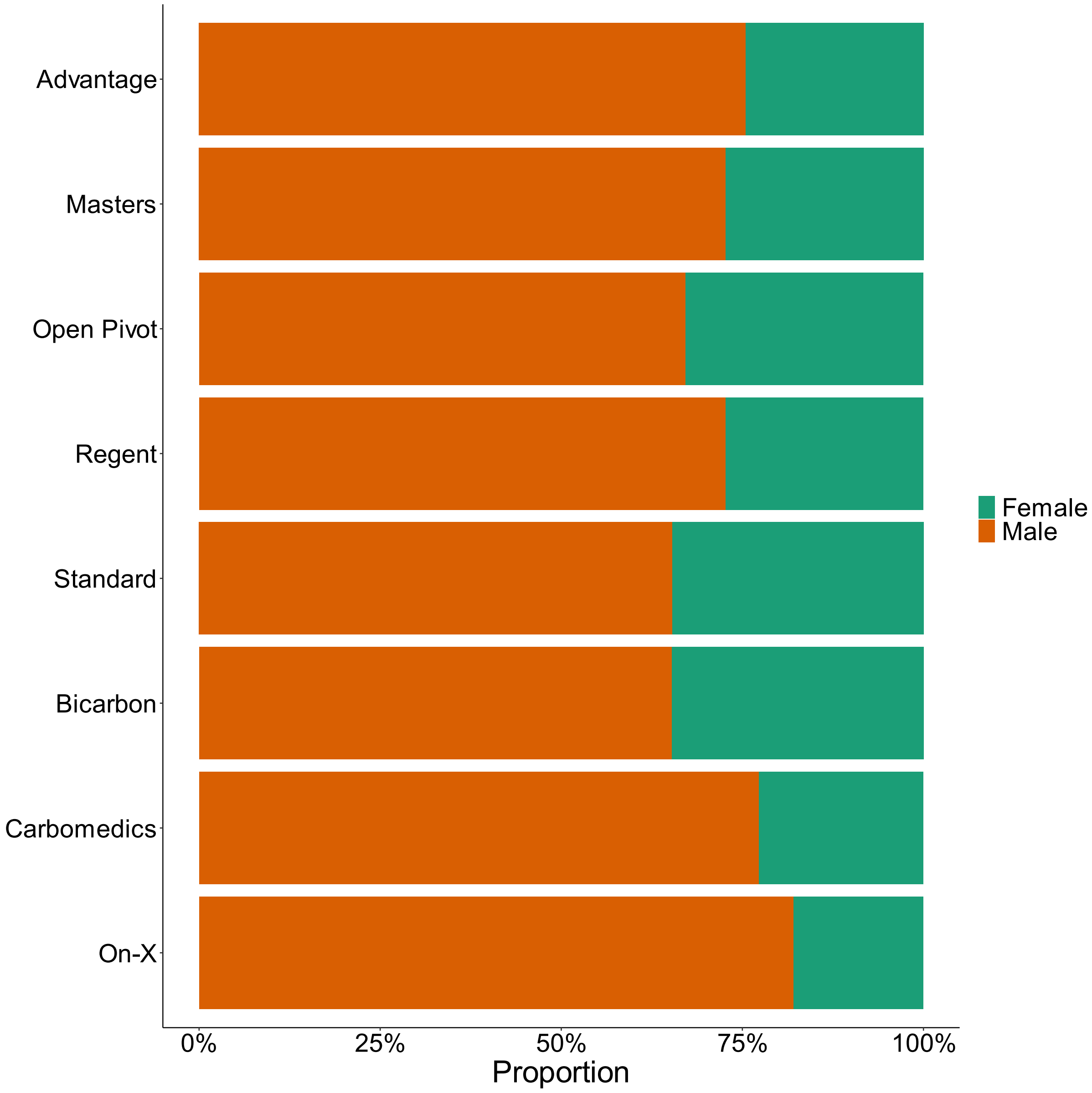

© 2024 Granbom Koski M et al. *JAMA Network Open*.

eFigure 6. The Regression Standardized Difference in Cumulative Incidence of All-Cause Mortality Following Surgical Aortic Valve Replacement With a Mechanical Valve Prosthesis in Sweden Between 2003 and 2018. A red curve indicates a statistically significant difference, i.e., a curve whose confidence interval does not cross over 0.

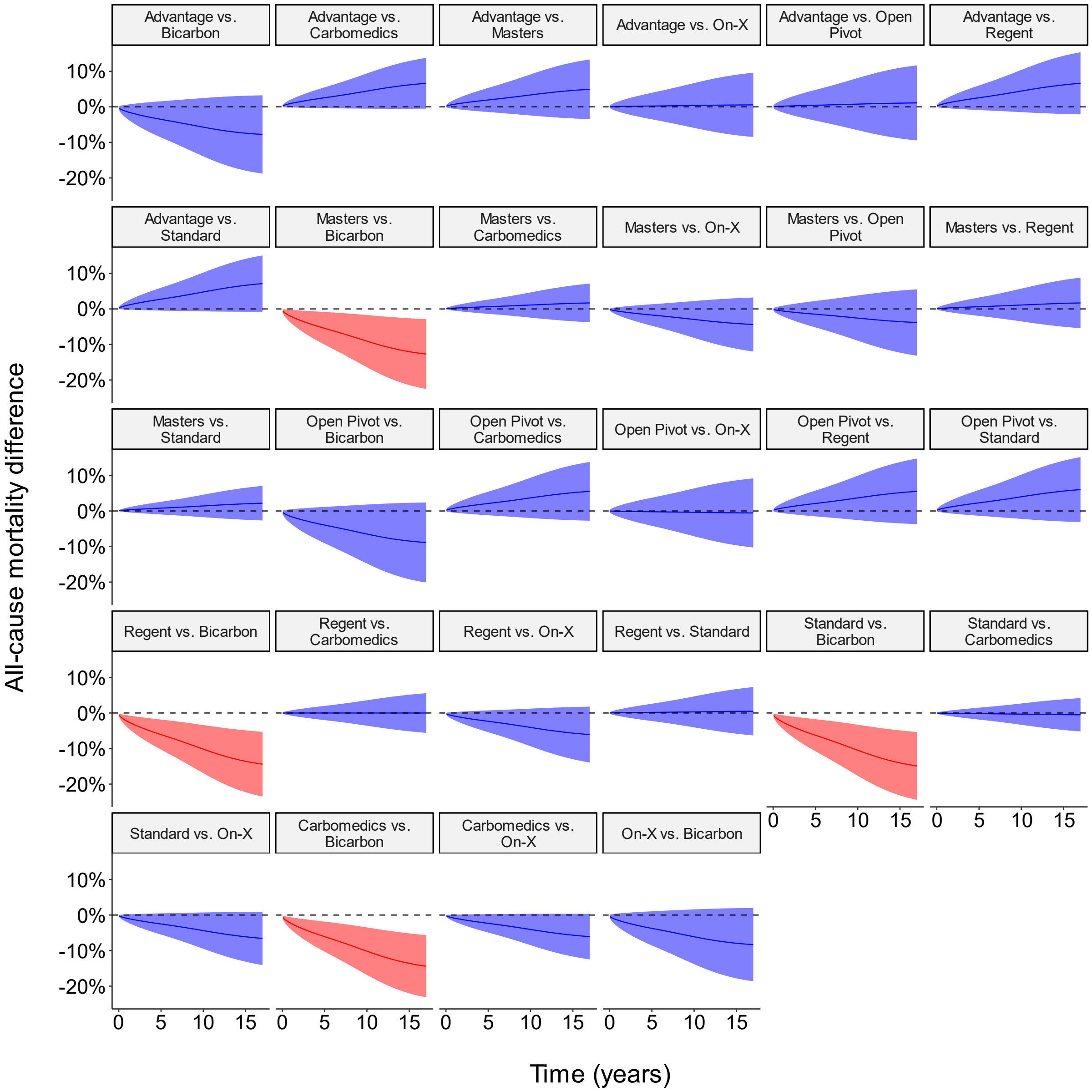

© 2024 Granbom Koski M et al. *JAMA Network Open*.

eFigure 7. The Regression Standardized Difference in Cumulative Incidence of Aortic Valve Reintervention Following Surgical Aortic Valve Replacement With a Mechanical Valve Prosthesis in Sweden Between 2003 and 2018. A red curve indicates a statistically significant difference, i.e., a curve whose confidence interval does not cross over 0.

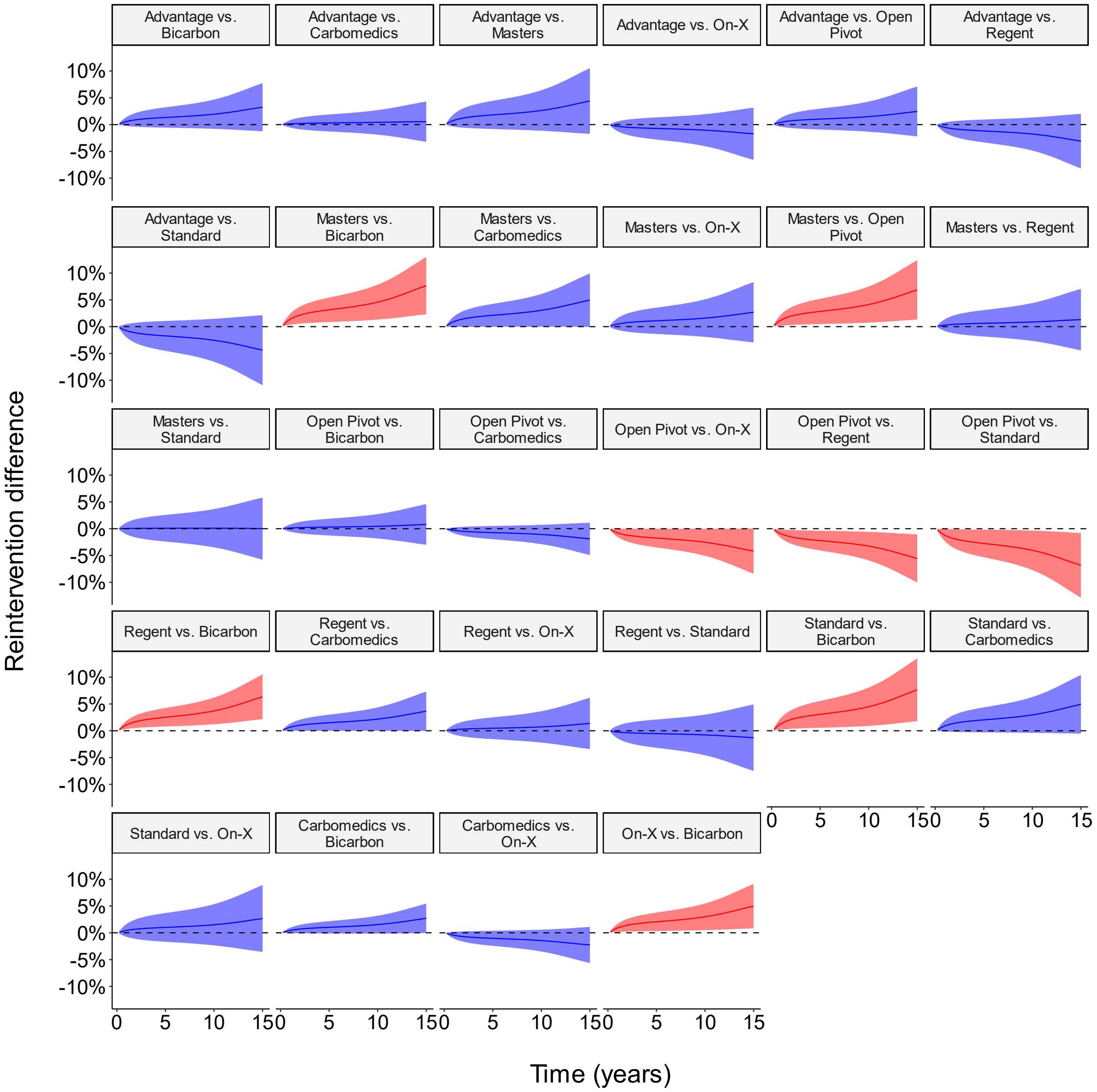

© 2024 Granbom Koski M et al. *JAMA Network Open*.

eFigure 8. The Regression Standardized Difference in Cumulative Incidence of Heart Failure Hospitalization Following Surgical Aortic Valve Replacement With a Mechanical Valve Prosthesis in Sweden Between 2003 and 2018. A red curve indicates a statistically significant difference, i.e., a curve whose confidence interval does not cross over 0.

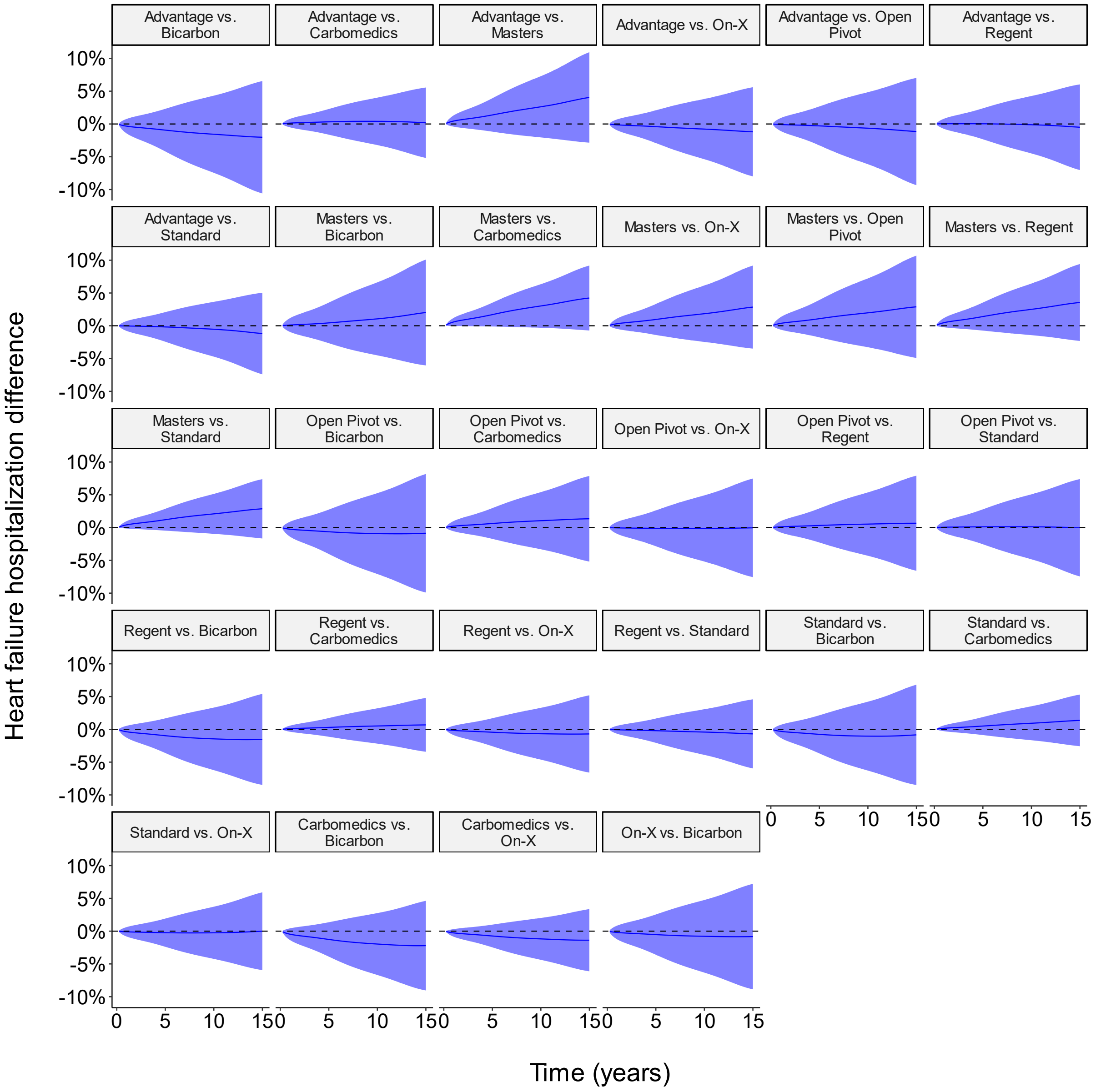

© 2024 Granbom Koski M et al. *JAMA Network Open*.

eFigure 9. The Regression Standardized Difference in Cumulative Incidence of Bleeding Events Following Surgical Aortic Valve Replacement With a Mechanical Valve Prosthesis in Sweden Between 2003 and 2018. A red curve indicates a statistically significant difference, i.e., a curve whose confidence interval does not cross over 0.

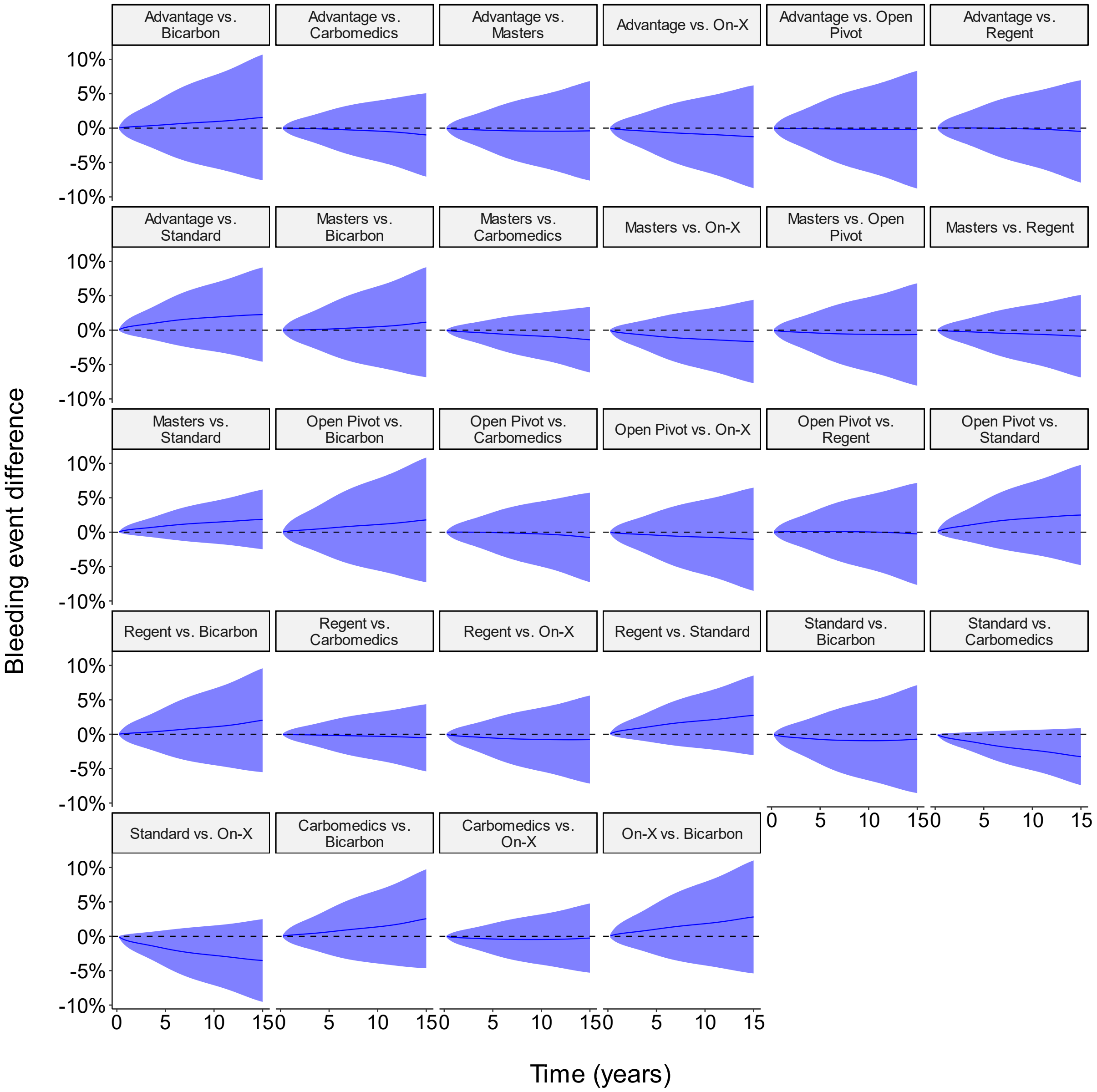

© 2024 Granbom Koski M et al. *JAMA Network Open*.

eFigure 10. The Regression Standardized Difference in Cumulative Incidence of Stroke, TIA or Embolic Event Following Surgical Aortic Valve Replacement With a Mechanical Valve Prosthesis in Sweden Between 2003 and 2018. A red curve indicates a statistically significant difference, i.e., a curve whose confidence interval does not cross over 0.

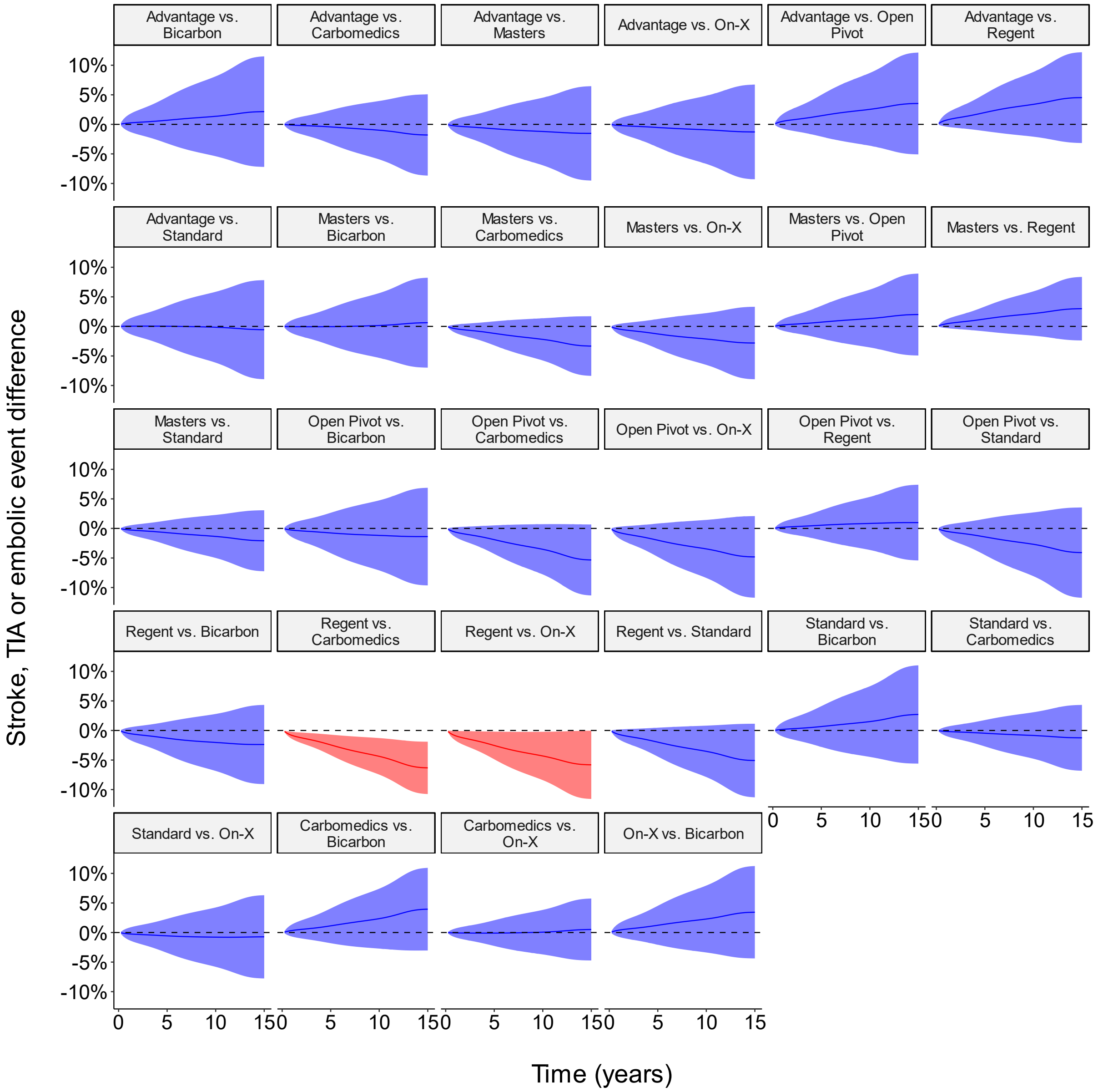

© 2024 Granbom Koski M et al. *JAMA Network Open*.

eFigure 11. Kaplan-Meier Estimated Survival and Number at Risk After Surgical Aortic Valve Replacement With Different Mechanical Valve Model Groups in Sweden Between 2003 and 2018

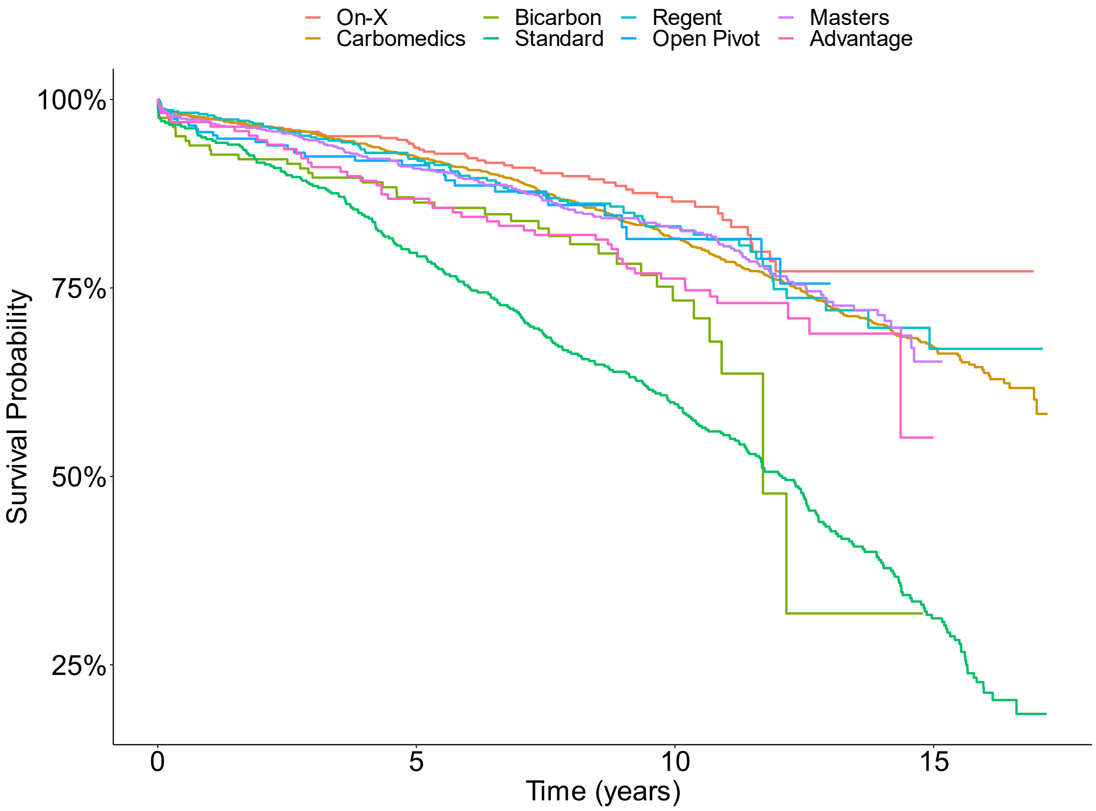

Number at risk

	0	5	10	15
On-X	679	360	146	6
Carbomedics	2171	1625	1008	312
Bicarbon	164	125	40	0
Standard	418	333	249	68
Regent	567	346	161	23
Open Pivot	231	143	46	0
Masters	827	618	364	17
Advantage	167	145	103	1

© 2024 Granbom Koski M et al. *JAMA Network Open*.

eFigure 12. Aalen-Johansen Estimated Aortic Valve Reintervention and Number at Risk After Surgical Aortic Valve Replacement With Different Mechanical Valve Model Groups in Sweden Between 2003 and 2018

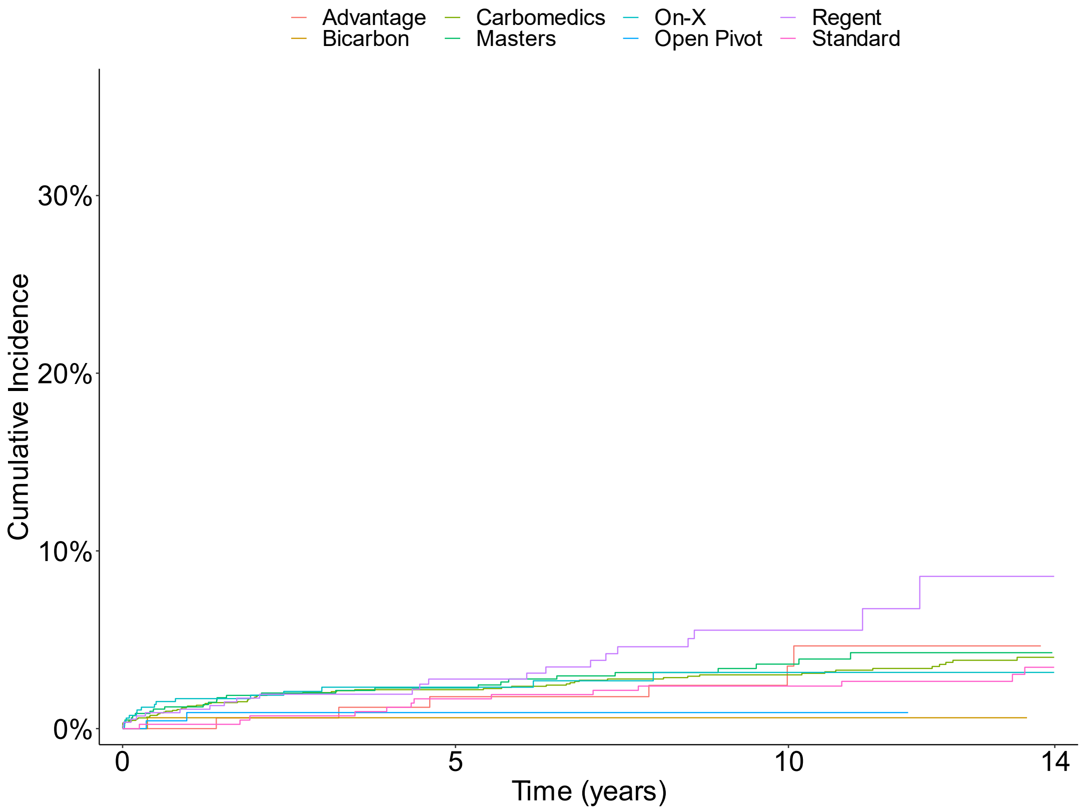

Number at risk

	0	5	10	14
Advantage	167	142	65	0
Bicarbon	164	104	9	0
Carbomedics	2171	1496	919	281
Masters	827	559	282	0
On-X	679	300	84	5
Open Pivot	231	115	35	0
Regent	567	289	105	19
Standard	418	327	231	78

© 2024 Granbom Koski M et al. *JAMA Network Open*.

eFigure 13. Aalen-Johansen Estimated Heart Failure Hospitalization and Number at Risk After Surgical Aortic Valve Replacement With Different Mechanical Valve Model Groups in Sweden Between 2003 and 2018

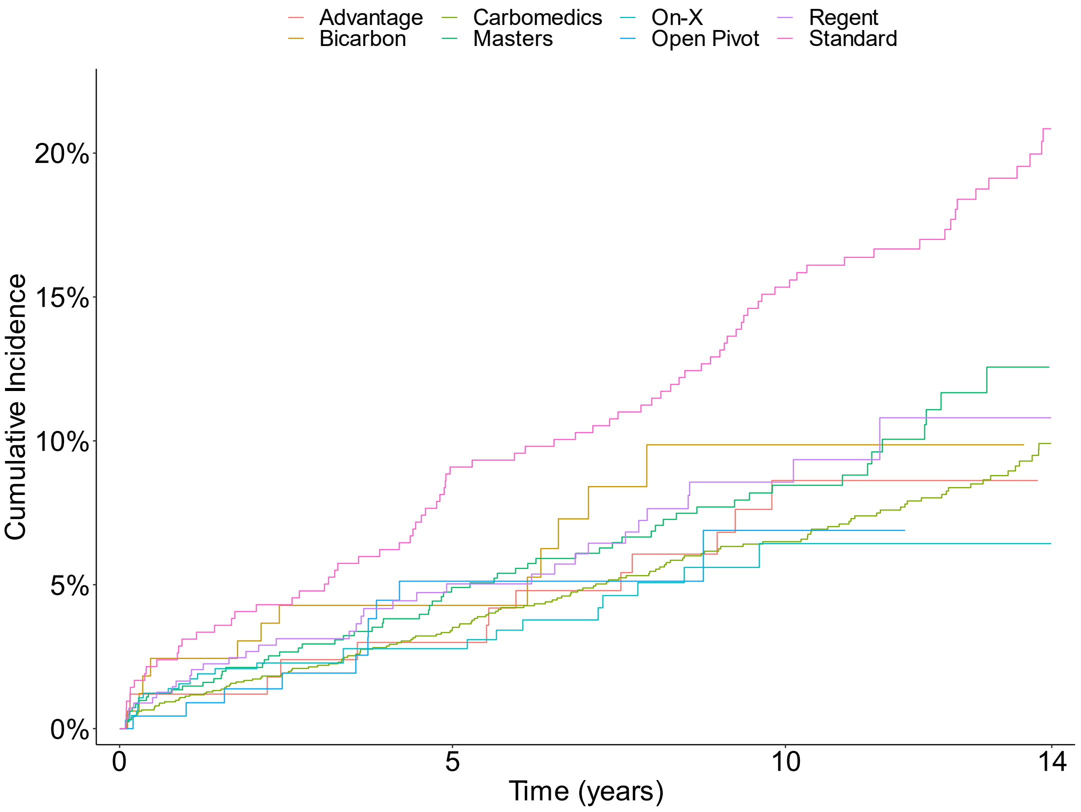

Number at risk

	0	5	10	14
Advantage	167	141	62	0
Bicarbon	164	101	8	0
Carbomedics	2171	1483	906	278
Masters	827	546	271	0
On-X	679	298	81	5
Open Pivot	231	110	33	0
Regent	567	281	100	20
Standard	418	307	214	68

© 2024 Granbom Koski M et al. *JAMA Network Open*.

eFigure 14. Aalen-Johansen Estimated Major Bleeding Event and Number at Risk After Surgical Aortic Valve Replacement With Different Mechanical Valve Model Groups in Sweden Between 2003 and 2018

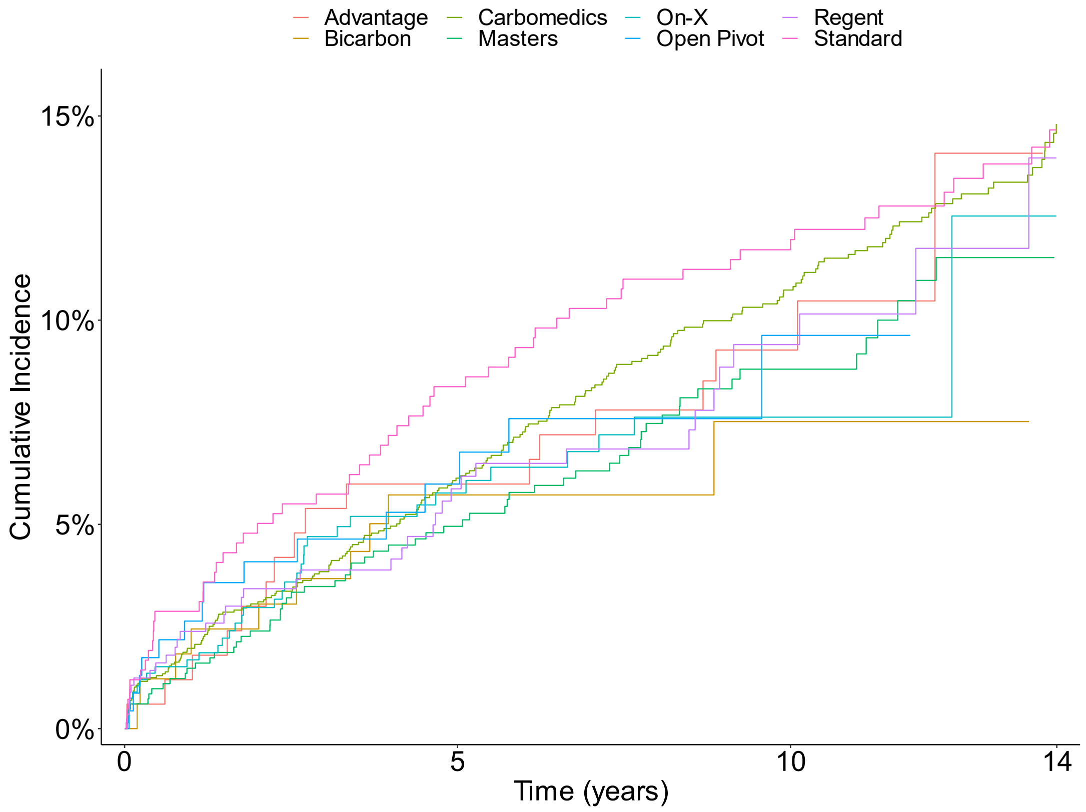

Number at risk

	0	5	10	14
Advantage	167	138	61	0
Bicarbon	164	101	8	0
Carbomedics	2171	1440	859	255
Masters	827	546	262	0
On-X	679	290	80	5
Open Pivot	231	108	34	0
Regent	567	284	104	19
Standard	418	306	212	70

© 2024 Granbom Koski M et al. *JAMA Network Open*.

eFigure 15. Aalen-Johansen Estimated Stroke, TIA or Embolic Event and Number at Risk After Surgical Aortic Valve Replacement With Different Mechanical Valve Model Groups in Sweden Between 2003 and 2018

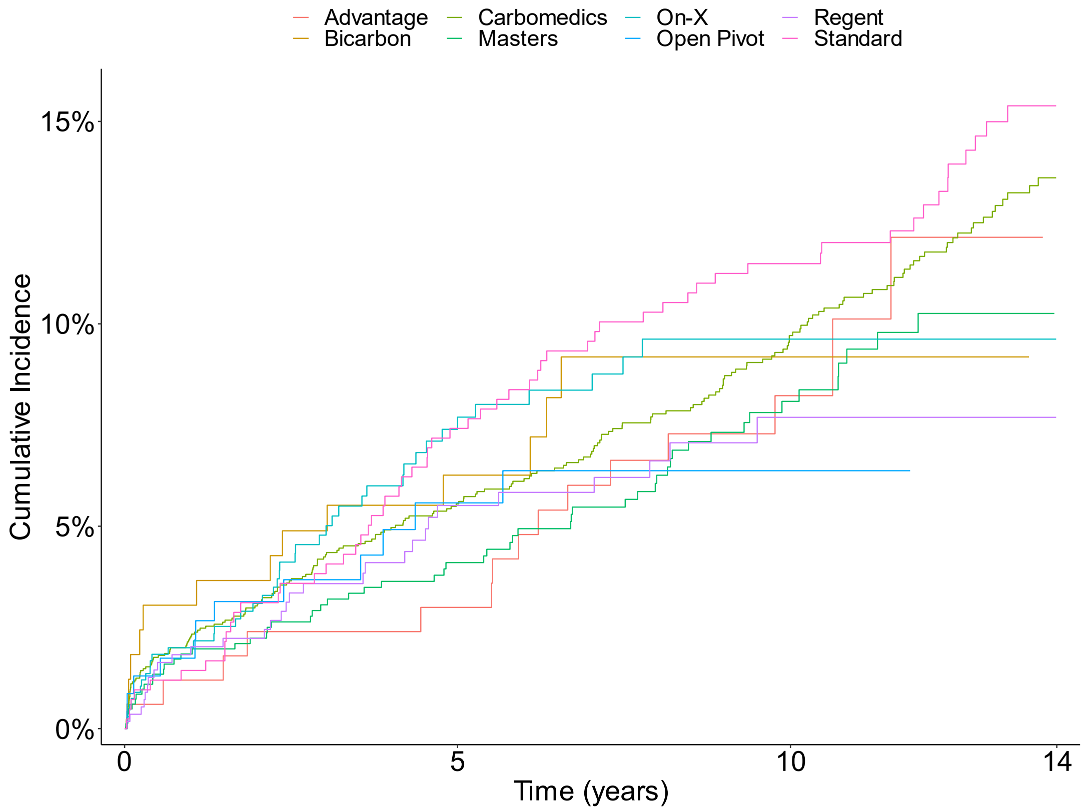

Number at risk

	0	5	10	14
Advantage	167	141	63	0
Bicarbon	164	100	9	0
Carbomedics	2171	1446	872	256
Masters	827	549	265	0
On-X	679	284	77	5
Open Pivot	231	111	34	0
Regent	567	282	99	19
Standard	418	308	211	71

© 2024 Granbom Koski M et al. *JAMA Network Open*.
