# Supplementary material for: Comparative Long-Term Clinical Performance of Mechanical Aortic Valve Prostheses
Source: JAMA Netw Open. 2024 Apr 19;7(4):e247525. doi: 10.1001/jamanetworkopen.2024.7525 (PMC11031681; doi:10.1001/jamanetworkopen.2024.7525)
Supplement: Supplement 2. — Data Sharing Statement [file jamanetwopen-e247525-s002.pdf]

Data Sharing Statement

Granbom Koski. Comparative Long-Term Clinical Performance of Mechanical Aortic Valve Prostheses. *JAMA Netw Open*. Published April 19, 2024.
doi:10.1001/jamanetworkopen.2024.7525

Data
Data available: No

Additional Information
Explanation for why data not available: Not allowed under Swedish law. It is however possibly to apply for data from the different governing bodies.
